# Rotational Dynamics of Linkers in Metal–Organic Frameworks

**DOI:** 10.3390/nano9030330

**Published:** 2019-03-02

**Authors:** Adrian Gonzalez-Nelson, François-Xavier Coudert, Monique A. van der Veen

**Affiliations:** 1 Catalysis Engineering, Department of Chemical Engineering, Delft University of Technology, 2629 Delft, The Netherlands; 2 DPI, P.O. Box 902, 5600 AX Eindhoven, The Netherlands; 3 Chimie ParisTech, PSL University, CNRS, Institut de Recherche de Chimie Paris, 75005 Paris, France; fx.coudert@chimieparistech.psl.eu

**Keywords:** metal–organic frameworks, linker dynamics, rotation, gate-opening effect, ^2^H NMR, computational chemistry

## Abstract

Among the numerous fascinating properties of metal–organic frameworks (MOFs), their rotational dynamics is perhaps one of the most intriguing, with clear consequences for adsorption and separation of molecules, as well as for optical and mechanical properties. A closer look at the rotational mobility in MOF linkers reveals that it is not only a considerably widespread phenomenon, but also a fairly diverse one. Still, the impact of these dynamics is often understated. In this review, we address the various mechanisms of linker rotation reported in the growing collection of literature, followed by a highlight of the methods currently used in their study, and we conclude with the impacts that such dynamics have on existing and future applications.

## 1. Introduction

Metal–organic frameworks (MOFs) are ordered arrays of polytopic organic ligands, commonly called linkers, interconnecting metal-based inorganic building units via coordination bonds. The assembly of a large number of organic and inorganic building units is what enables a vast array of topologically diverse frameworks [1]. In particular, the choice and design of the organic linkers is an equally important means of endowing these crystalline materials with specific desired capabilities. Often, these organic components confer some type of flexibility, including rotational mobility [2]. Rotational motion in MOFs is a contrasting characteristic with respect to other ordered microporous materials, such as zeolites, which contain no intrinsic rotor components and are especially challenging to functionalize.

Rotor MOFs can be considered a subset of amphidynamic crystals: solids that combine high order with high molecular mobility [3]. In conventional amphidynamic crystals, molecular rotors are assembled in the crystal lattice via non-covalent interactions, and they usually must contain bulky groups that act as spacers to create enough free volume for rotation to occur. In MOFs, however, these needs are overcome as a result of the porosity that is attained via highly directional coordination bonds. 

Rotational dynamics is bound to influence several aspects of MOF properties, perhaps most importantly their interaction with guest molecules. As an example, rotational mobility plays a central role in the fascinating behavior of the zeolitic imidazolate framework ZIF-8 [4,5]. The linkers in this framework are known to aid in the diffusion of surprisingly large guest molecules through the otherwise small micropores, by swinging, or partially rotating. This gate-opening, as we shall see, occurs in a considerable variety of different rotor MOFs.

Additionally, the possibility to manufacture tailor-made molecular rotors in an ordered scaffold opens up a multitude of options for novel applications, such as sensors, stimuli-responsive materials [6], and crystalline molecular machines [7].

In the last years, an increasing number of papers have appeared that elucidate this type of flexibility in MOFs [8,9,10,11,12]. So far, no review on rotational linker dynamics of MOFs exists. Yet, rotation appears to be a rather common type of dynamics in this class of materials, with clear consequences for adsorption and separation of molecules, optical, and mechanical properties. This review begins by addressing the variety of linker rotational motions and their mechanisms (Section 2), followed by a highlight of the methods currently used in their study—including experimental and computational (Section 3), and finalizing with the impacts of such dynamics on MOF applications (Section 4).

## 2. Types of Rotational Linker Dynamics

Michl and coworkers [13] conceptualized rotational motions in molecules by defining them as rotor systems analogous to macroscopic machines:

“Molecular rotor: a molecular system in which a molecule or part of a molecule rotates against another part of the molecule

Rotator: the part of the molecule or system that rotates against the rest

Stator: the stationary part of the system with respect to which the rotator turns

Axle: the portion of the molecule that carries the rotator and about which the rotator turns”

**Scheme 1:**
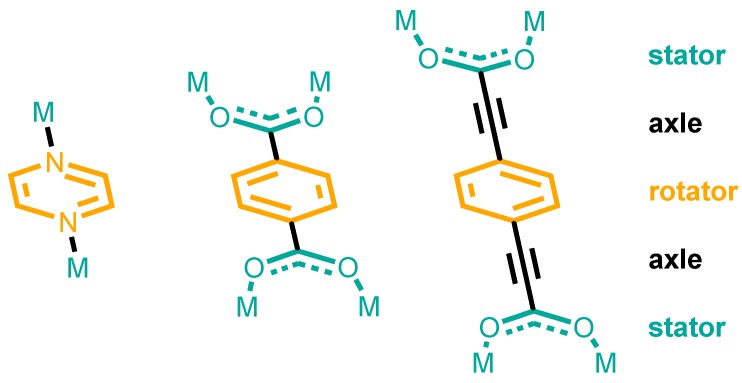
Common types of axles in metal–organic framework (MOF) rotor linkers.

For the purpose of this review, we have adapted these definitions to metal–organic frameworks in a manner that will allow us to cover all relevant cases to the best of our knowledge. These definitions were proposed for the broader context of molecular rotors, yet they can be easily applied to metal–organic frameworks when we consider that the inorganic building units, together with the functional groups coordinated thereto, form the stator. Common axles, shown in Scheme 1, can be composed of one or more bonds, for example: the nitrogen–metal coordination bond, the covalent bond between the benzene ring and the carboxylate, or the ethynyl (–C≡C–) group.

**Figure 1:**
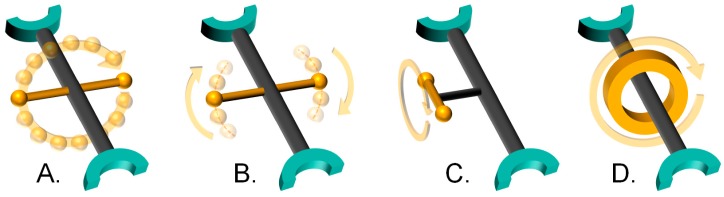
Illustration of the four types of rotational linker dynamics: (**A**). Complete rotation, (**B**). Partial rotation, (**C**). Side group rotation, (**D**). Mechanically interlocked molecule rotation.

Within this frame, we categorized rotational dynamics of MOF linkers into four groups (also represented in Figure 1):

- Complete rotation
- Partial rotation
- Rotation of side groups
- Mechanically interlocked molecule rotation

The first three types of rotation have a common ground in that their axles are covalent bonds. Type A closely resembles the typical case of molecular rotors described by Michl et al., where the axle crosses (approximately) the center of mass of the rotator, and connects it to two stators opposed to each other. We group in this category all rotors that are able to complete 360° torsions.

**Figure 2:**
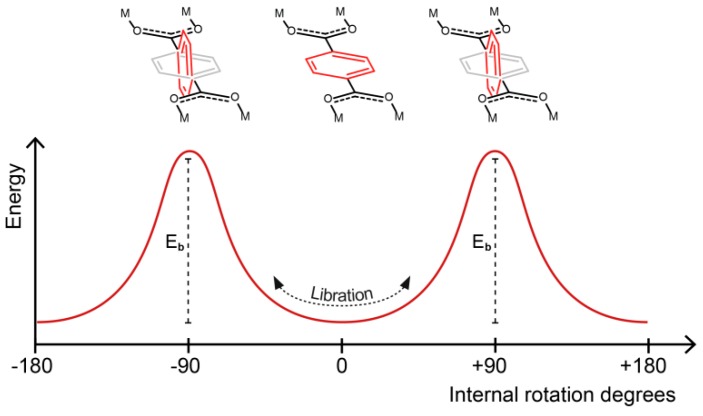
Torsional potential curve of a terephthalate rotor.

Complete rotation often occurs in steps comprising a fraction of the 360° path, which are often referred to as jumps, hops or flips. The nature of these jumps is determined by the torsional potential of the rotor [13]. Figure 2 illustrates the torsional potential of a terephthalate rotor. It can be seen that minima in the potential energy are found every 180° of relative rotation between the *para*-phenylene rotator and the coordinated carboxylate stators. These minima determine the starting and ending position of the jumps, which must surpass an energetic barrier, *E*_b_.

It is important to note that the jump angles will vary depending on symmetry and structural factors. Furthermore, when the thermal energy of the system is much higher than the energetic barrier, the rotor will rotate freely instead of performing discrete jumps, a scenario that is known as free or diffusional rotation [10].

Type B rotation involves rotational motions that do not lead to full rotations. We have separated these from Type A since they can be considered a special case where the rotor is greatly hindered, either due to intrinsic or extrinsic restrictions, and 360° rotations do not occur. We can make an important division within this group:

- dynamics where the rotor performs rotational motions about a minimum in a potential energy well, or torsional potential minimum, which are called *librations*.
- dynamics where the rotor overcomes a maximum in potential energy and reaches a second conformation. For the sake of clarity, they will be referred to as *hops* in this section.

Librations are formally torsional vibrational modes, and as such their frequencies are largely determined by the shape of the potential well, while only their amplitude depends on thermal energy [13]. It should be understood that systems that present Type A motions may also exhibit Type B; for example, when a rotator undergoes librations as well as complete rotation. Librations of the phenylene rotator are shown in Figure 2 as the arrow centered on a local minimum, representing small-angle partial rotations without exceeding the torsional barrier. Hops, in contrast, do involve a thermally activated transition from one local minimum to another, in the same fashion as already described in Type A, with the condition that they do not achieve 360° rotations.

Due to these fundamental differences, librational and hopping dynamics fall in entirely different timescales. Still, they can in principle be related to each other in a molecular rotor. The librational frequency of a rotor may be considered approximately equal to the attempt frequency [13], or pre-exponential factor, in the equation describing the rate of a thermally activated rotational hop:

(1) $$\mathrm{rate}=\omega_{0}e^{\frac{-E_{b}}{kT}},$$

where ω_0_ is the attempt frequency, *E*_b_ is the energetic barrier for rotation, k is the Boltzmann constant, and *T* is the temperature.

It should be noted that the occurrence of rotational hopping entails the existence of librations, although the latter are often not detected. The inverse relation does not hold.

Type C will involve the few reported studies of meta-rotors, or rotors within MOF linkers. The defining characteristic of this group is that the axle and rotator do not play a role in the framework’s connectivity; they are a side group on the linking struts. Therefore, the main linker component can be considered the stator, with the functional group attached to it via a covalent bond “axle”.

Type D rotation involves systems where the rotator is a separate molecule that is mechanically interlocked to the axle. Here, only weak interactions—such as dispersion forces and hydrogen bonds—are the basis of contact between the two.

### 2.1. Type A: Complete Rotation

A list of rotor systems discussed in this sub-section can be found in Table 1, together with the relevant information regarding rotation mechanism and energy barriers. We will begin our review of Type A rotations with arguably the most common type of rotor in the MOF field: terephthalate (or 1,4-benzenedicarboxylate; 1,4-BDC) linkers.

The first account of complete rotational motion of linkers in MOFs was published by Gonzalez et al [14], where the dynamics of the phenylene units in the quintessential MOF-5 were examined. The authors transferred the use of solid-state deuterium nuclear magnetic resonance (NMR), which had so far been used to probe the dynamics of molecules within zeolites, microporous silica, and polymer backbones [15], to the direct measurement of the dynamics of the perdeuterated phenylene in the terephthalate linkers. It was found that the phenylene groups are static at room temperature (within the timescale of the experiment, i.e., slower than ~ 10^3^ Hz), and that they undergo fast 180° rotations (π-flips) at 373 K. These results were later confirmed by Gould et al. [16], who used the same two-fold exchange model to fit the ^2^H NMR data, and applied the Arrhenius relation to assign an activation energy of 47.3 ± 8.4 kJ mol^−1^. This experimental activation energy corroborated the barrier of 52 kJ mol^−1^ that had been predicted earlier for this framework using DFT (density functional theory) calculations [17], where the 90° twist of the benzene ring with respect to the carboxylate plane was indeed found to be the maximum in the potential energy curve.

It should be remarked that the MOF-5 structure exerts little steric hindrance on the rotating phenylene rings [16], and therefore the relatively high rotational barrier can be mainly attributed to electronic effects. That is, the planar conformation of the terephthalate is stabilized through π electron delocalization, and a 90° rotation of the phenylene implies a complete loss of π system delocalization between the benzene ring and the carboxylate planes [10].

The same mechanism of complete rotation has been observed in several other terephthalate-based MOFs, including the well-known MIL-47(V), MIL-53 [18,19,20], and UiO-66 [8,21,22] families. The rotational barriers for all these systems are within 30–50 kJ mol^−1^, regardless of the metal centers, which suggests the barrier is in fact intrinsically determined by the linker, with some variations due to metal electronegativity and possible steric effects from the crystal lattice.

Similar findings were reported for the terephthalate rotor in the M_2_(1,4-BDC)_2_(DABCO) pillared MOFs (also known as DMOF series) that consist of 2-D sheets of divalent metal ions (M = Co^2+^, Zn^2+^, Ni^2+^, and Cu^2+^) in the paddlewheel secondary building unit (SBU) linked by terephthalate and pillared by 1,4-diazabicyclo[2.2.2]octane (DABCO) to a 3D structure. With ^2^H NMR it was also shown that the phenylene groups performed π-flipping motions that were largely independent of which metal cations were present [23]. The activation energies derived from this ^2^H NMR study were in the range of 32–36 kJ mol^−1^.

Analysis of terephthalate rotors has been extended to cases where the pores are occupied with guest molecules. The presence of molecules in the pores is expected to have a significant effect on the mobility of the linkers lining the pores due to weak interactions as well as steric effects. In the case of MIL-53(Al), the effect of xylene loading was analyzed and found to hinder the phenylene π flips greatly, increasing the activation energy from 37 to above 50 kJ mol^−1^ [19]. The π-flipping of UiO-66(Zr) was found to have a strong linear dependence on benzene loading, increasing from 30 kJ mol^−1^ in the guest-free framework, to 48 kJ mol^−1^ with the highest loading of molecules [21]. Likewise, in the experimental studies of unfunctionalized DMOF systems described above, the presence of *N*,*N*-dimethylformamide inside the pores increased the activation energy from 32–36 to 47–55 kJ mol^−1^ [23].

In the Zn-DMOF family, DFT calculations were also employed to model the rotational energy barrier for terephthalate linkers with a series of functionalizations, as well as for 1,4-naphthalenedicarboxylate (1,4-NDC) [30]. Using fragment models to approximate the MOF structure, the potential energy surface was scanned while rotating the phenylene group. Drastic differences in estimated rotational barriers were found when analyzing different substituent groups on the benzene ring, ranging from 10 to 58 kJ mol^−1^. The authors attributed the decrease in barrier for rotation to the electrostatic repulsion between electronegative atoms as ring substituents and the oxygen atoms of the carboxylate groups, yet the electron donating/withdrawing effect of the groups was not considered.

**Figure 3:**
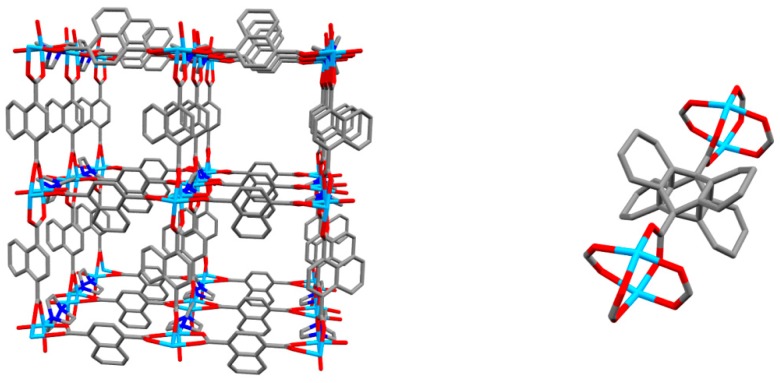
Structure of pillared DMOF with 1,4-NDC rotors. Adapted with permission from [26]. Copyright Wiley-VCH, 2006.

Only the barriers for terephthalate and 1,4-NDC (58 and 22 kJ mol^−1^, respectively) could be compared to available experimental data; the calculation of the terephthalate resulted in an overestimation the rotational barrier of 36 kJ mol^−1^ reported by Khudozhitkov et al. [23]., while for 1,4-NDC the model underestimated the 53 kJ mol^−1^ found previously by Horike et al. [26]. The latter ^2^H NMR study assumed four-site jumps of the 1,4-NDC rotor, based on their observation of four disorder positions of the rotator in the single crystal X-ray diffraction (XRD) structure (Figure 3). 

Regardless of the substantial underestimation of the barrier of 1,4-NDC, the computational study confirmed the presence of two maxima in the torsional potential of this rotor, one at 0° and the other at 90° with respect to the carboxylate plane. The energy wells between these maxima in a 360° rotation correspond to the four sites that were used to model the ^2^H NMR spectra.

**Figure 4:**
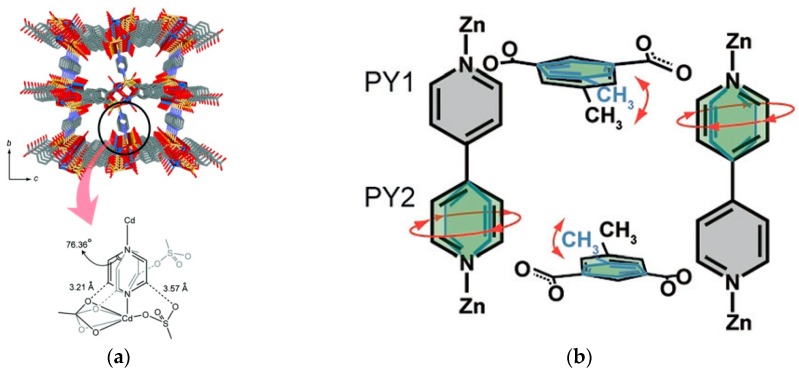
Structure of two N-linker rotor-based MOFs. (**a**) CdNa(2-stp)(pyz)_0.5_(H_2_O), with an illustration of the two disorder positions of the linker; (**b**) 2D stacked framework, Zn(5-Me-isophthalate)(bipyridine). Reproduced with permission from [26] and [31]. Copyright Wiley-VCH, 2006. Copyright Wiley-VCH, 2018.

Horike et al. also showed that rotational dynamics occur in linkers that are not dicarboxylates [26]. Initially, single crystal XRD studies indicated an equally disordered pyrazine ring over two positions, suggesting that this ligand completes a full 360° rotation in four steps, or jumps, around the N–N axis (precisely at 0°, 76.4°, 180°, and 256.4°) (Figure 4a). Therefore, in contrast with the terephthalate rotor π-flip cases, a four-site jump model was used to fit the ^2^H NMR spectra, and an activation energy of 7.7 kJ mol^−1^ was derived. 

Such a striking difference in energetic barriers for rotation between this rotor and the various phenylene-based systems is not surprising. The fact the axle of the pyrazine rotator is a σ bond with no double bond character implies that this type of coordination will exert less electronic limitations than the terephthalate rotors, which have a certain double bond character in the C–C axle due to π electron delocalization.

It is interesting to note that only one activation energy was determined, even though the two sets of jumps used for analysis are geometrically unequal (two short jumps and two large jumps), suggesting the possibility of two barriers. Therefore, it could be inferred that the jumps are either energetically degenerate, or that the differences are too small to be detected.

Inukai et al. have researched dynamics of a different type of two-linker 2D stacked framework material, Zn(5-X-isophthalate)(bipyridine), where X stands for methyl, nitro, or methoxy groups [31,32]. These systems were studied with ^2^H NMR to describe the rotational dynamics of each type of ligand. The 4,4’-bipyridine pillar’s dynamics were found to be fairly complex (see Figure 4b), and dependent on the functional group used on the dicarboxylic linker. With the presence of methyl and nitro groups, one of the rings of the bipyridine was measured to be static, since the functional group of the isophthalate greatly hinders its rotation. The other pyridine ring, however, performed full rotations through a combination of π-flips and 4-site jumps. Unfortunately, no activation energies were reported, and thus no comparison may be made with respect to other N-donor ligands.

The use of 5-methoxyisophthalate, in contrast, brought about enough steric hindrance to block the rotation of both pyridine rings simultaneously. The dynamics of the angled dicarboxylate linker are an interesting example of incomplete rotations and will be addressed in Type B.

**Figure 5:**
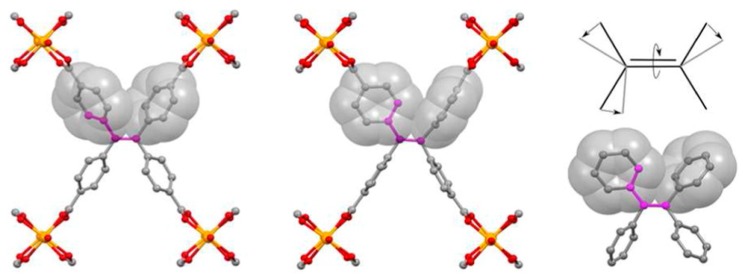
Linker conformation in tetra(4-carboxy)phenylethylene-based MOF, depicting steric hindrance from neighboring rings (left and center). The comparative case of a free tetraphenylethylene molecule is shown on the right. Reproduced with permission from [25]. Copyright American Chemical Society, 2012.

Shustova et al. investigated phenylene dynamics in a tetra(4-carboxy)phenylethylene-based MOF [25], whose linker structure is depicted in Figure 5. A combined ^2^H solid-state NMR and DFT study revealed that the phenylene rotators undergo rotations via π-flips, with an activation energy of 43 kJ mol^−1^. Although such a high barrier is within the range of terephthalate rotors that we have discussed previously, a comparison of the computed rotational barrier in model molecules (including styrene, benzoic acid, and tetraphenylethylene) led the authors to conclude that, in this case, an important fraction of the rotational barrier is of steric origin. Since the four arms of the linker are tethered to metal nodes, the core is barred from deformations that would otherwise allow for one phenylene to rotate with less interaction with its neighbor (as was observed in the free tetraphenylethylene molecule; right side of Figure 5). 

**Figure 6:**
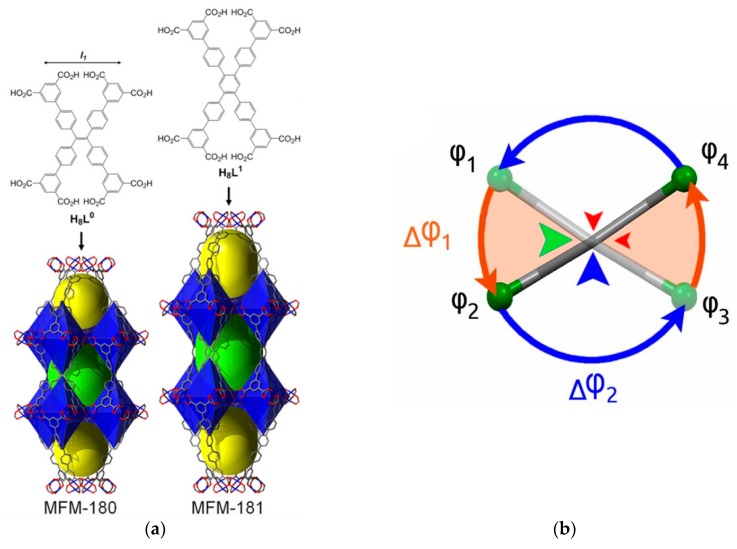
(**a**) Structure of MFM-180 and MFM-181 and their respective linkers; (**b**) rotational trajectory of the *para*-phenylene rotators. Reproduced from [9].

A case of four site jump rotation of phenylene groups was recently reported in two octacarboxylate frameworks, MFM-180 and MFM-181 (Figure 6a) [9]. Due to their branched structure, both linkers contain only one type of rotator that can undergo complete rotations: a *p*-phenylene in each arm connecting the core of the linker (ethylenyl or benzene ring, respectively) with the benzenedicarboxylate terminal rings. 

Detailed analysis of the ^2^H NMR line shape evolution along a wide temperature range allowed the authors to conclude that the mechanism for complete rotation involved a four-site jump-exchange in both MOFs. The four jumps, however, are not equal, since two different rate constants could be derived from the NMR data. In both cases, one of the jumps involves a smaller angle partial rotation and is even activated at 100 K (Δφ_1_ jump angle indicated in Figure 6b with a green arrow), while a second, wider motion (Δφ_2_), was only activated at 330 K, allowing for full-rotational movement at these higher temperatures (see blue arrow in Figure 6b). Because of the phenylene group’s C_2_ symmetry, Δφ_1_ and Δφ_2_ occur twice within a complete 360° rotation.

**Figure 7:**
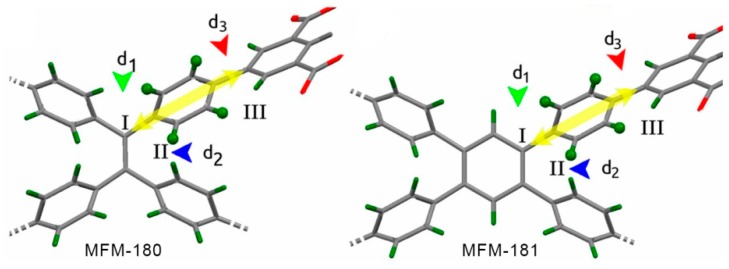
Rotator environment in MFM-180 and MFM-181. The colored arrows mark the closest steric interactions. Reproduced from [9].

A closer look at each linker’s structure and the possible interactions of the rotating phenylene with neighboring rings revealed the likely origins of such a complex rotational behavior. On the one hand, the smaller barrier for Δφ_1_ was attributed to steric hindrance from the site marked with a green arrow in Figure 7, where the structural differences explain the large difference in Δφ_1_ jump rates between both MOFs. The large barrier for Δφ_2_, on the other hand, which is activated at approximately 330 K in both frameworks, was proposed to originate from the interactions with the neighbor ring in the vicinity, marked with a blue arrow in Figure 7, which is similar in both MOFs.

**Figure 8:**
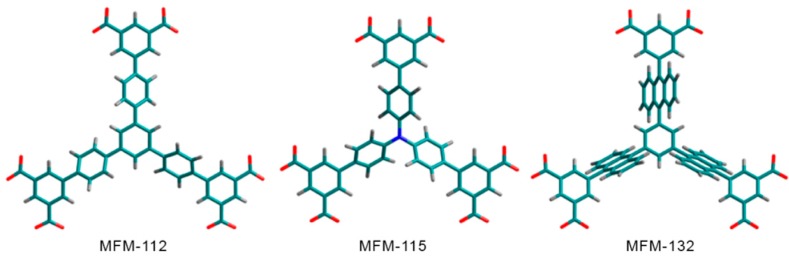
Structure of rotor linkers used in MFM-112, -115, and -132. Reproduced with permission from [12]. https://pubs.acs.org/doi/10.1021/jacs.7b05453; further permissions should be directed to the ACS. Copyright American Chemical Society, 2017.

Another branched linker MOF with complete rotational motions was studied in a similar fashion. Three different tri-branched, hexacarboxylate linkers were used to build isostructural frameworks [12]. Two of the members of this series were found to perform full rotations (Figure 8, left and center). The third ligand’s athracenylene rotors (Figure 8, right), in contrast, could not achieve complete rotations due to its highly hindered environment, and will be discussed in Type B rotations. MFM-112 and MFM-115 differ in that the linker of the former has a benzene ring core, while the latter has a nitrogen core connecting the three dicarboxylate-terminated branches. These systems closely resemble the MFM-180 and -181 previously discussed in that the rotator is a phenylene group connecting the terminal ring to the core of the linker. Likewise, these rotors undergo four-site jumps to complete a 360° rotation.

The authors determined that the *p*-phenylene groups in MFM-115 require significantly more thermal energy to rotate than in MFM-112, which should be expected due to the shorter distances between rotators owing to the smaller center group. Additionally, two different modes of rotation were observed in MFM-115: a slow rotation with a barrier of 14 kJ mol^−1^ below 283 K, and a faster motion with higher energy barrier (40 kJ mol^−1^) above 283 K. The authors hypothesized that the low temperature motion corresponds to the collective, gearlike rotation of the three phenylene groups. It is feasible that concerted motion would result in the diminishment of steric hindrance, as well as lower rates of rotation. At higher temperatures, concerted motion becomes less likely, thus increasing the steric hindrance from neighboring rings leading to a higher energetic barrier (40 kJ mol^−1^).

**Figure 9:**
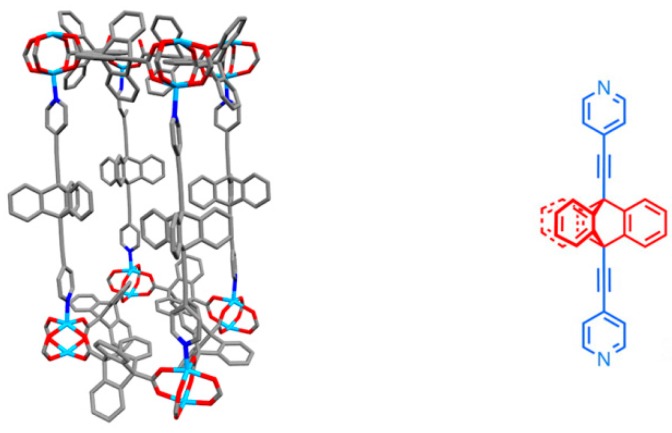
Crystal structure of UCLA-R3 (**left**); the triptycene rotator is shown in red (**right**). Adapted with permission from [33]. Copyright American Chemical Society, 2016.

Garcia-Garibay’s group reported a framework containing a triptycene rotator with triple bond axles in a Zn-based pillared architecture (see Figure 9) [33]. Although the triple bond axles were expected to lead to a free rotor system, solvent DMF (*N*,*N*-dimethylformamide) molecules present in the pores of the MOF were found to strongly hinder the rotation, leading to an apparent barrier of 56.5 kJ mol^−1^. 

**Scheme 2:**
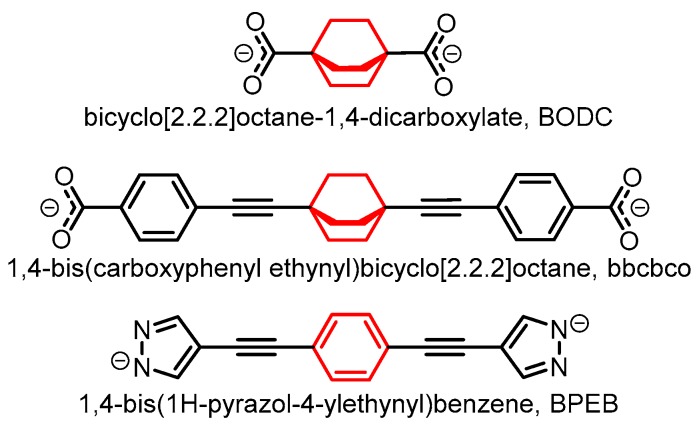
Low-barrier rotor linkers. Rotator fragments are shown in red.

Aiming to achieve lower rotational energy barriers in MOF rotor systems, Garcia-Garibay’s research group explored a ditopic linker analogous in connectivity length to the terephthalate, 1,4-bicyclo[2.2.2]octane dicarboxylate (BODC, see Scheme 2, top). BODC linkers, in contrast to terephthalates, present no π electron delocalization to provide a double bond character to the C–C bond between rotator and stator (carboxylate). Additionally, the combined symmetry of the C_3_ rotor (bicyclo[2.2.2]octane, BCO) and the C_2_ stators leads to sixfold degenerate energy potential, meaning that the rotor should be able to perform a full rotation in six jumps of equal activation energy. Altogether, it was demonstrated that the BODC rotors have much lower rotational barriers than terephthalates, emphasized by the fact that unrestricted diffusional rotation was observed down to ca. 20 K, although with increasing contributions of six-fold exchange. Below 13 K, three-fold rotation was observed, while at 6 K the slow exchange regime was detected (frequencies lower than 1 kHz). Since the rotation of the BCO groups was too fast to derive appropriate rate constants from line shape analysis, the authors performed ^2^H and ^1^H T_1_ relaxation measurements, which yielded activation energies of 0.5–0.77 kJ mol^−1^.

Another framework containing a bicyclo[2.2.2]octane rotator was constructed by Bastien et al. [28]. The remarkable length of the axle (linker depicted in Scheme 2, center) would have meant that all steric interactions might have been avoided, thus achieving free rotation. However, the framework was found to be interpenetrated, and therefore the expected low rotational barrier could not be achieved. A barrier of 15.5 kJ mol^−1^ was estimated computationally, mostly caused by steric hindrance from neighboring rotators.

A different approach to achieving free rotation in MOF linkers was presented by Bracco et al. [29], where a *p*-phenylene unit was selected as rotator with a triple bond axle coordinated to Zn^2+^ centers via pyrazole groups (Scheme 2, bottom). Using ^2^H NMR, very fast π-flips were observed down to 150 K, such that the rate constants of rotation could not be determined through line shape modeling. Instead, the ^1^H spin-lattice relaxation times were measured and the Arrhenius equation was used to determine an activation energy of 2 kJ mol^−1^.

Although the majority of cases reported in the literature involve rotation of phenylene groups in terephthalate linkers, several other cases illustrated here show that this type of motion is quite diverse. The rotational barriers associated to complete rotations in MOFs can be found in a wide range, from less than 1 kJ mol^−1^ to close to 50 kJ mol^−1^.

We can observe that the great diversity in rotor systems and their mechanisms of rotation arise mainly from variations in the electronic configuration of the rotor and the steric environment in frameworks. The location of maxima and minima along the torsional potential curve determine the partial steps that a rotor must follow, while the height of rotational barriers is related to the rate of rotation. In addition, guest molecules have been shown to significantly hinder rotational dynamics of linkers, a property of great importance for adsorption applications which will be addressed in Section 4.1.

### 2.2. Type B: Partial Rotation

Librational motions in MOFs often fall within the THz frequency region, and are thus detectable with spectroscopic techniques such as inelastic neutron scattering and far-infrared spectroscopy. The combination of detailed ^2^H NMR and spin-lattice relaxation analysis, which together can probe an enormous range of frequencies, have established that systems that perform complete rotations may also present librational motions within the same molecular rotator fragment, such as in the case of UiO-66 [8]. 

Soft modes attributed to libration of phenylene groups in other terephthalate-based frameworks have been discussed in several instances. In the case of MOF-5, this type of motion was initially identified using inelastic neutron scattering and ab initio calculations [17]. The well-known “breathing” MIL-53 was suspected to obtain its intriguing bi-stable pore configuration (narrow pore, large pore) as a result of these twisting modes [34].

**Figure 10:**
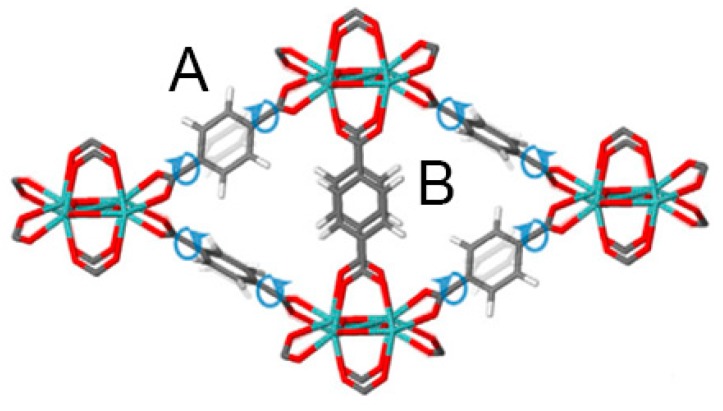
Structure of MIL-140A, terephthalate linkers in two different positions (A and B). Reproduced with permission from [24]. Copyright American Physical Society, 2017.

Similarly to MIL-53, the MIL-140A framework is built from inorganic 1D chains and terephthalate linkers. However, within the latter structure, there are two different environments for the linkers (A and B, see Figure 10). Using a combination of neutron scattering, far-infrared spectroscopy, and ab initio quantum chemical calculations, researchers unraveled the contributions of librational motions of phenylene groups in the low-frequency vibrational spectrum [24]. It was found that each type of linker had phenylene twisting modes at different frequencies, and that the B linker’s rotation required higher energies due to steric hindrance of the adjacent linkers. In fact, supporting single-point DFT calculations showed that complete rotation of the hindered B linker is likely impossible.

Revisiting the MFM hexacarboxylate series discussed in Type A, the third member that Yan and co-workers designed, MFM-132, contains a 9,10-anthracenylene moiety on each of the three arms (Figure 8, right). Their ^2^H NMR analysis demonstrated that these rotators were not able to perform full rotations even at high temperatures (ca. 573 K) due to the steric hindrance from adjacent groups, yet a small amplitude libration of 32° was observed. 

**Figure 11:**
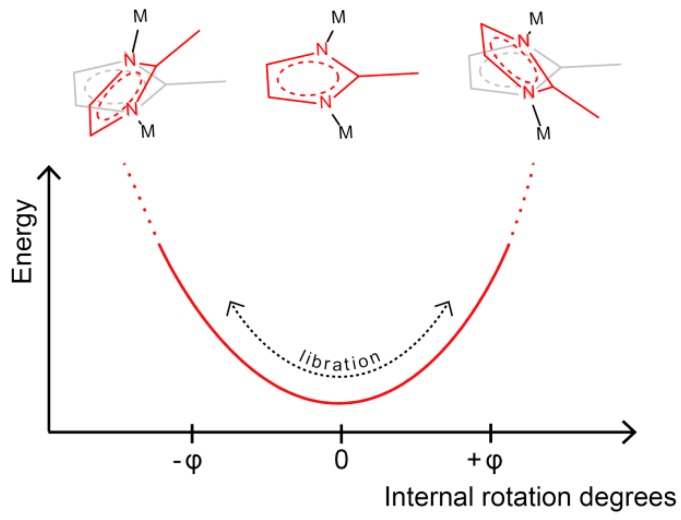
Illustration of the torsional potential for coordinated imidazolate linkers (as in zeolitic imidazolate frameworks (ZIFs)). Large amplitude rotations are hampered by the connectivity angle, yet small-amplitude librations are possible.

In some instances, the impediment to rotate is a result of a ligand coordination geometry that deviates from 180°. Although not as many such cases have been reported, a remarkable example is found in zeolitic imidazolate frameworks (ZIFs). An imidazolate ligand’s bridging coordination angle is approximately 145°, which means any large amplitude rotations will be strongly hampered by the directionality of the coordination bond (see Figure 11). However, ample evidence has shown that even small amplitude rotational motions are responsible for the remarkable capacity of MOFs such as ZIF-8 to admit molecules that are seemingly too large to fit through the pore windows [35]. 

**Figure 12:**
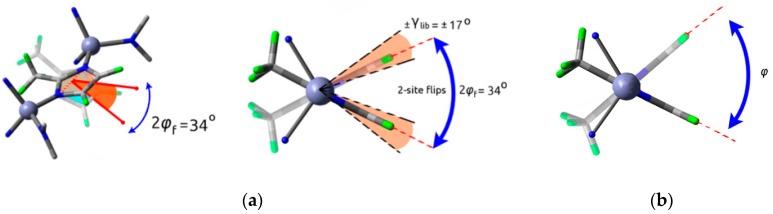
Diverse dynamics of 2-methylimidazolate linker in ZIF-8. (**a**) Small-amplitude two site hopping (34°) and fast librations (± 17°) centered on both hop positions; (**b**) slow, large-amplitude swing. Adapted with permission from [36]. https://pubs.acs.org/doi/10.1021/acs.jpcc.5b09312; further permissions should be directed to the ACS. Copyright American Chemical Society, 2015.

The rotational dynamics of the 2-methylimidazolate linker have been studied in detail using ^2^H NMR [11,36], and multiple modes of rotation have been identified, including fast, small-angle librations (shaded in orange in Figure 12a), small-amplitude two site hopping (blue arrows in Figure 12a), and slow, large-amplitude swinging between two sites (Figure 12b). In the case of ZIFs, the term swinging has been used to describe partial rotational motions indistinctly, even though they may have different characteristics (e.g., librations or hops). Strikingly different energy barriers have been assigned to the latter two rotational motions: 1.5 kJ mol^−1^ and 50–60 kJ mol^−1^, respectively. The torsional potential is thus certainly more complex than what is described in Figure 11, likely with local minima at different torsion angles.

**Scheme 3:**
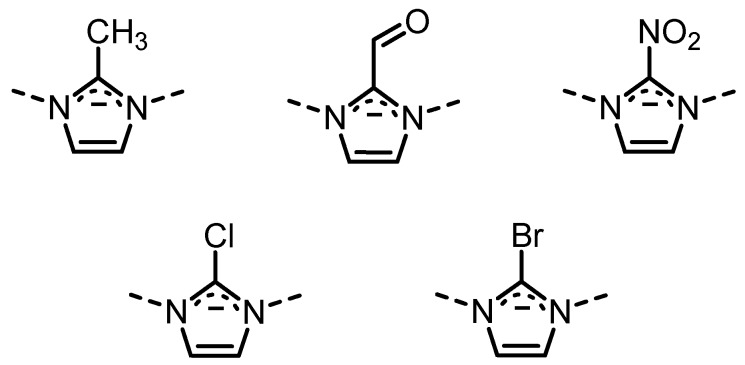
Imidazolate linkers used in ZIF-8 isoreticular frameworks.

Structural transitions in isostructural ZIFs have been studied to understand their behavior upon molecule uptake, taking into consideration the effect that the choice of functional group (methyl, carboxaldehyde, and nitro, shown in Scheme 3) has on the dynamic responses [37]. Researchers have found that, when subjected to alcohol guest molecules under high hydrostatic pressures, the framework responds by adopting a high-pressure conformation where the imidazolate linkers swing to varying degrees depending on the functional group chosen to substitute the 2- position of the ring. This collective swinging motion leads to changes in pore volume and pore window diameters, with the methyl- and aldehyde-containing linkers this leads to a larger pore window, while the nitro-functionalized linkers rotate in the opposite direction, leading to a smaller pore window.

Interest in the effect of functionalization on the imidazolate linker’s rotational flexibility has motivated a recent study comparing the behavior of ZIF-8 with its 2-Cl and 2-Br functionalized isoreticular analogues [38]. Using first-principles molecular dynamics, the authors found that the 2-chloroimidazolate linkers responded to N_2_ adsorption by swinging in a similar manner as the 2-methylimidazolate, while the bromo-substituted linker does not rotate, showing that the framework is more rigid. It was proposed that the bulkiness of the bromo group, and not its electronic effects on the coordination bond, is accountable for this lack of flexibility.

Inukai and coworkers developed a MOF that combines both full rotation of pyridine rings (already discussed in Type A rotation) and libration of a secondary linker [31]. The 5-methylisophthalate group resembles the previously discussed imidazolate linker in that the connectivity is not linear, but angular (120° in this case). Due to this, 1,3-phenylenes cannot perform complete rotations, yet they can still have rotational dynamics in the form of librations. ^2^H NMR evidence showed that the linker in this system indeed librates, aiding in guest molecule transport by widening the pore window.

The discussed cases of partial rotational motions should provide evidence that this is a broad category of dynamics, encompassing low-energy vibrational rotations that are expected to occur in any linker with rotational degrees of freedom, and hindered, thermally activated hops along local minima in the torsional potential. In contrast with full rotations, partial rotations have been documented in frameworks with angular linkers. In particular, the importance of this type of dynamics in frameworks such as the prominent ZIF-8 will be highlighted in Section 4.

### 2.3. Type C: Rotation of Side Groups

A third type of rotational motion occurring in the linker consists in the rotation of a side group that is covalently bonded to the main structural linker. Although a large variety of cases exist where these motions are, in principle, possible, there are only a handful of reported cases.

From the systems discussed in the previous sections, those involving a methyl group attached to the linker, methylimidazolate in ZIF-8 and 5-methylisophthalate in [Zn(5-Me-ip)(bpy)], are a clear example in this category. In both cases, methyl C_3_ rotations around the C–C bond were observed in the ^2^H NMR line shapes. Methyl rotation has a very low activation barrier estimated at 0.6–0.8 kJ mol^−1^ in ZIF-8 [39,40], therefore these fast motions are omnipresent in these systems in all covered temperature ranges [31,36], and can be detected in the THz range of the vibrational spectrum.

In ZIF-8, methyl rotations are not as accountable for guest molecule transport via gate-opening as are the librations of the whole imidazolate linker. Yet one can imagine that the impact of functional group rotation would increase for bulkier or more complex groups. ZIF-90, an isostructural framework to ZIF-8, the rotation carboxaldehyde group about its axis on position 2 was observed computationally to have an important effect on the angle of the imidazolate linker due to intra-framework interactions with neighboring linkers [41]. In other words, the librational angle is much smaller for ZIF-90 than for ZIF-8. Hence, the authors postulated a driving mechanism where the functional group rotation enhances or restricts the linker swing motion, which would likely have an effect on the gas adsorption process.

IRMOF-3, the amine-functionalized version of MOF-5, was subject of investigations regarding its linker dynamics [42]. The authors succeeded in using ^1^H NMR relaxation measurements to assign a second, lower-energy process besides the ring π-flip: the rotation of the amine group. Since the amine group participates in hydrogen bonding with the nearby carboxylate oxygen, a complex interplay between the amine rotation and the complete rotations of the phenylene may be expected.

Rotation of linker functional groups adds an additional aspect of design in MOFs. Although few cases have been reported so far, the further study of MOF linker dynamics should unearth more interesting examples in the future with potentially interesting effects on applications.

### 2.4. Type D: Rotation of Mechanically Interlocked Molecules

Metal–organic rotaxane frameworks (MORFs) are a relatively young class of MOF. This type of framework originates from the interest in endowing rotaxanes with a higher degree of order, as their functions had, until recently, mainly been studied in solution [43]. Yaghi and Stoddart [44] highlighted MORFs as central in the exciting prospect of obtaining frameworks capable of dynamics that do not compromise the structural units (i.e., the linkers). Since the rotator and stator are not covalently bonded in this type of rotor systems, supramolecular interactions between the ring and the threading component, as well as between the ring and the surrounding environment, should determine the rotational dynamics.

**Figure 13:**
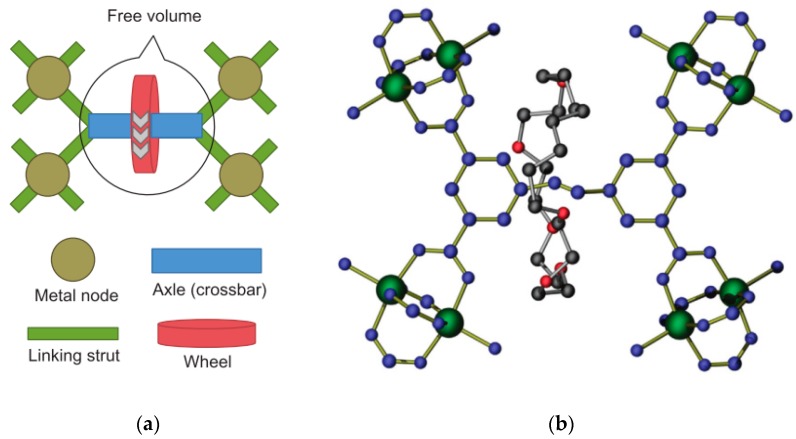
(**a**) Illustration of the composition of a metal–organic rotaxane framework (MORF); (**b**) Crystal structure of the Cu(II) MORF with a [2]rotaxane. Reproduced with permission from [45]. Copyright Nature Publishing Group, 2012.

The first report of rotational dynamics of a [2]rotaxane linker in a MOF came in 2012 [45], were the wheel component mechanically interlocked around a carboxylate functionalized axle in a Cu(II)-based MOF was found to transition from a static state to a thermally activated rotational state at 324 K (Figure 13). The fact that rotation was only possible in the activated framework (that is, guest molecule-free) provides evidence of the importance of MOF porosity in achieving dynamic MORFs. In a following study [46], a different MORF based on the same wheel component ([24]crown-6, or 24C6 macrocycle), a solvation-induced structural transition was also found to provide a means to control the rotation of the wheel about the linker axle. 

A comparative study that considered three different macrocyclic components of varying sizes, which imply different degrees of free volume allowing for ring dynamics [47]. It was found that only the wheel components without a benzo group ([22]crown-6 and [24]crown-6) were able to perform rotational motions, out of which only the larger wheel could rotate completely around the axle. Another report suggested the variation of weak interactions between wheel and axle components as a rotation control parameter [48]. These results have elucidated the remarkable degree of control that can be achieved through the rational design of rotating moieties in MORFs.

## 3. Methods for the Study of Linker Dynamics

### 3.1. Solid-State Nuclear Magnetic Resonance (NMR)

The numerous examples of complex linker dynamics in MOFs covered in Section 2 evidences the fact that ^2^H solid-state NMR is the leading experimental technique in MOF rotational dynamics research. Deuterium NMR is an outstanding technique to analyze molecular dynamics in the solid state for several reasons. Firstly, since deuterium has a nuclear spin of 1, its NMR properties are largely determined by quadrupolar interactions with the electric field gradient tensor [49]. The electric field gradient on an organic compound deuteron is determined predominantly by the electrons in the C–^2^H bond, therefore axial symmetry along this bond can be assumed [50]. This allows for dynamics to be tracked by analyzing the orientation of individual C–^2^H bonds [15,51]. 

It should be noted that natural abundance of the deuterium isotope is too low (0.0156%) [51], making the use of ^2^H-labeled rotator groups compulsory. This is often not a substantial drawback, as simple deuterated linker precursors are commercially available, but it may potentially deter the study of more complex linker architectures. An obvious benefit, however, is that ^2^H NMR is highly selective due to this labelling [15].

In most of the cases presented in this review, solid echo pulse sequences were used. In some instances, the use of other pulse sequences may allow to probe an extended frequency range. Spin alignment echo technique, for example, allowed Khudozhitkov et al. to probe rotational motions of *p*-phenylene below 1 kHz rate [20], which is the typical lower limit of conventional solid echo experiments.

**Figure 14:**
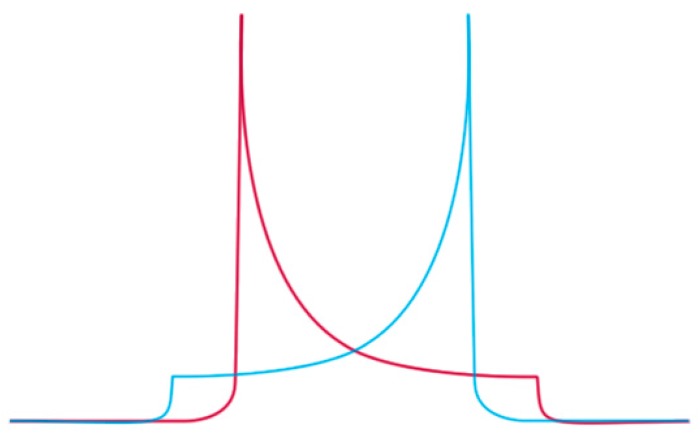
Pake pattern, displaying the two line shapes that compose it.

The spin 1 nucleus of deuterium has three Zeeman energy levels, therefore, its spectra contain two transitions. The splitting between these transitions is determined by the orientation of the C–^2^H bond with respect to the external magnetic field. Since a powder sample contains all possible orientations of C–^2^H bonds, the static ^2^H solid-state NMR spectrum of a powder sample has a characteristic broad, mirrored shape with two horns, called Pake (or powder) pattern [15]. Each half of the line shape is caused by one of the transitions (see Figure 14), in combination to all the possible orientations of the bonds. The maximum (the so-called horn), corresponds to the signal when the angle between the C–^2^H bond and external magnetic field is 90°. The shoulders of the line shape correspond to the 0° orientation, and are the least probable orientation in a powder sample.

**Figure 15:**
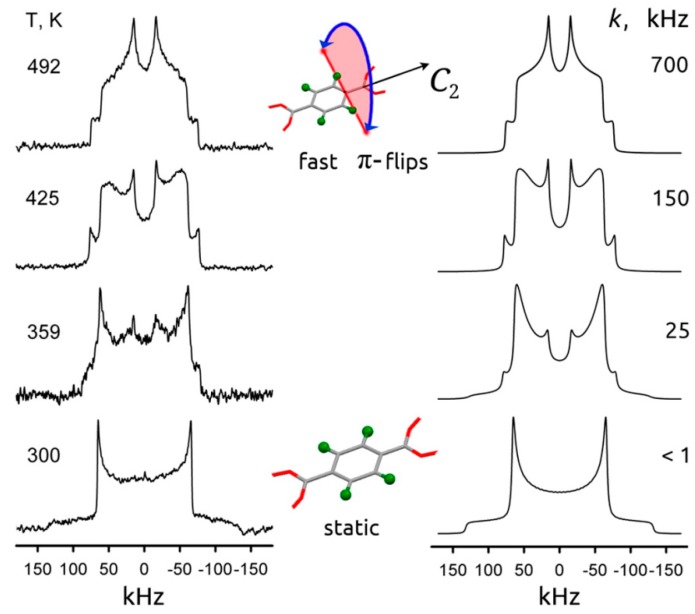
Experimental (**left** column) and simulated (**right** column) ^2^H NMR line shapes of the *p*-phenylene rotor in MIL-53(Al) at different temperatures. Reproduced with permission from [19]. Copyright American Chemical Society, 2014.

The Pake pattern can be highly sensitive to molecular motions that result in reorientation of the C–^2^H bonds. The frequency and trajectory of these variations are the main factors that will determine the distortions on the Pake pattern [52]. As an example, the left column of Figure 15 shows the evolution of the ^2^H NMR line shape with temperature in deuterium-labelled MIL-53(Al). A two-site π-flip model was used to model the spectra, based on the known symmetry and behavior of terephthalate rotors. The good agreement between the frequency-fitted line shapes (right column of Figure 15) and the experimental spectra confirms that the distortions of the Pake pattern are in fact a result of the gradual increase in frequency of π-flipping motions. This example demonstrates that modeling the line shape with respect to possible trajectories is an essential part of analyzing molecular dynamics using ^2^H solid-state NMR spectroscopy.

The frequency range of ^2^H NMR measurements can be conveniently divided into three dynamic frequency regions, corresponding to their effects on the Pake pattern: the slow motion limit (SML), for rates of 1 kHz or less; the intermediate motion regime (IMR), when rates are between 10^3^ and 10^7^ Hz; and the fast motion limit (FML), when rates are higher than 10^7^ Hz [47].

Molecular dynamics of C–^2^H bonds that fall in the SML and in the FML regions do not cause further changes in the Pake pattern line shape. Therefore, not much information can be gathered from processes occurring at these frequencies. Indeed, the SML line shape can be modeled with a single set of parameters. In the case of FML line shapes, the complete trajectory of motion must be known.

Dynamics occurring in the IMR, in contrast, cause marked differences in the line shape depending on the geometry of the different deuteron sites, and on the exchange rate between them. Spectra are thus collected at varying temperatures along the range, and the different spectral line shapes must be fitted to a dynamic model. Such a model includes, among other information, the parameters that describe the motion’s trajectory through exchange sites, and the exchange rate. 

**Figure 16:**
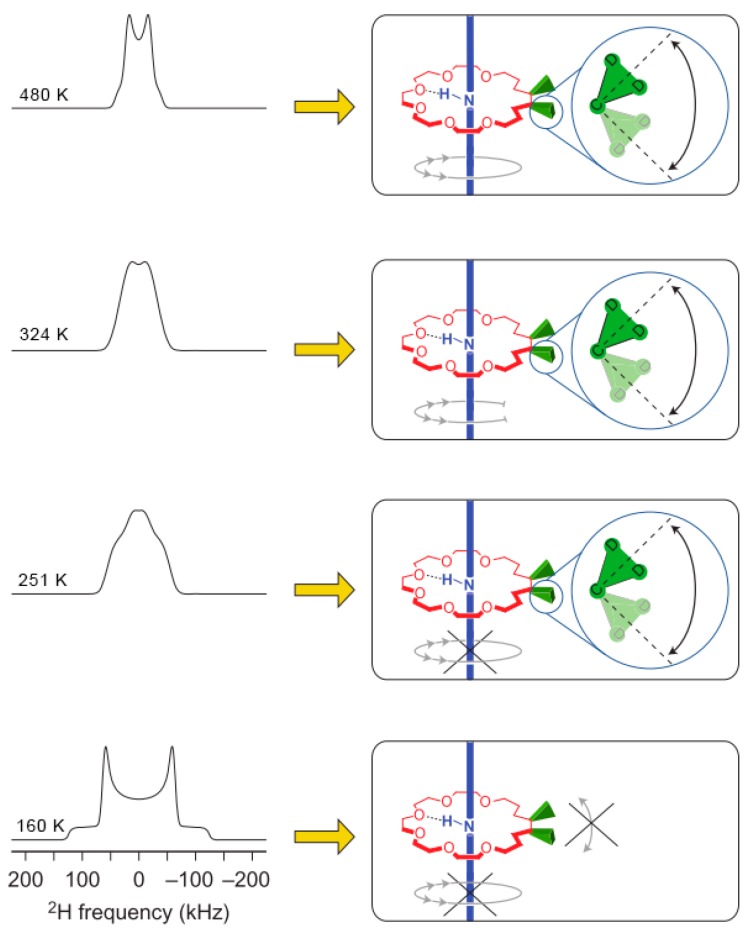
Simulated ^2^H NMR line shapes of the metal–organic rotaxane framework UWDM-1 at different temperatures. The corresponding models are shown on the right-hand side. Reproduced with permission from [45]. Copyright Nature Publishing Group, 2012.

The complexity of deuterium NMR line shape analysis may increase when the studied systems have more degrees of freedom in the C–^2^H bonds, or when more than one type of deuterium bond is present. The work by Schurko and Loeb’s groups on rotaxane MOFs [45] illustrates one such case (see Figure 16). With the help of detailed models, several modes of motion could be identified at different temperatures, including the CD_2_ group reorientation, as well as partial and complete rotations of the macrocycle around the axle component.

**Figure 17:**
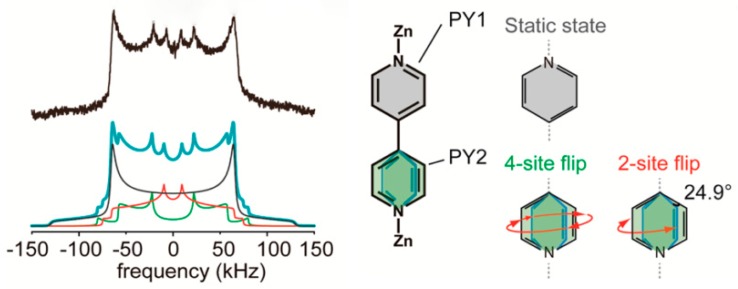
Experimental (top spectrum) and simulated (bottom spectra) ^2^H NMR line shapes. The corresponding models for each of the three rotational situations are shown on the right-hand side. Reproduced with permission from [32]. Copyright American Chemical Society, 2015.

The work by Inukai et al. [32] depicts another interesting case of complexity solved by deuterium NMR, where two pyridyl rings (PY1 and PY2 in Figure 17) behave differently depending on their position in the crystal lattice. Good agreement with the experimental line shape (black line on the left of Figure 17) was reached by modeling a superposition of the line shapes of the PY1 static ring (gray line shape), and the mobile PY2 ring performing 4-site and 2-site flips (ring shown in green, with the 4-site line in green and the 2-site line in red). Similar modeling was implemented in several other publications addressed in Section 2 [12,19,26,33]. 

Besides deuterium NMR, some examples of ^1^H NMR used to study linker rotational motions in MOFs can be found. Morris et al. [42] analyzed ^1^H spin-lattice relaxation (T_1_) data obtained from IRMOF-3, a framework containing aminoterephthalate linkers. Two different motions were identified: a low-energy motion (activation energy 5.0–7.5 kJ mol^−1^) and a higher energy process with an activation energy of 21 kJ mol^−1^. Based on the frequency behavior these processes, the authors postulated that the low-energy motion should be a libration, and the high-energy motion a π-flipping motion.

^1^H NMR spin-lattice relaxation analysis was further applied in two studies involving ultrafast rotation in MOFs. Bracco et al. [29] carried out these measurements on the Zn-BPEB framework to complement ^2^H NMR line shape analysis that revealed dynamics belonging to the fast motion limit (>10^7^ Hz). Indeed, the rotational frequency at 150 K was found to be in the order of 10^11^ Hz, and a fitting of the Kubo–Tomita relation led to an activation energy of 2 kJ mol^−1^. 

Vogelsberg et al. [10] measured T_1_ spin-lattice relaxation times down to much lower temperatures (2.3–80 K) in the BODCA-MOF. They found a single process in the MHz frequency range, which could be fitted to the Kubo–Tomita equation, yielding an activation energy of 0.77 kJ mol^−1^. This motion was confirmed to be the rotation of the BCO moiety by means of ^2^H NMR experiments previously addressed in Section 2.

More recently, Damron et al. [53] introduced the use of a different NMR method to study linker dynamics in MOFs: a separated local field (SLF) type known as DIPSHIFT (which stands for dipolar chemical shift correlation). This technique was applied to a series of the UiO-66 family, and differences in rotational motions could be inferred from the variations in ^13^C–^1^H dipolar coupling strength. The analysis relies on the fact that, with faster motions of the benzene ring, the ^13^C–^1^H dipolar coupling constant decreases. It was thus found that the dimethyl functionalization vastly increased the rotational mobility of the linker in the MOF, while the presence of hydroxyl groups on the ring had the opposite effect.

In conclusion, solid-state NMR offers an interesting variety of approaches to study molecular dynamics in solids. Deuterium NMR clearly stands out in the field of MOFs due to the unparalleled level of detail that can be drawn from its analysis, especially when trying to discern between different types of rotation. The other types of NMR spectroscopy here discussed offer the advantage of different frequency ranges, and do not require chemical labelling. However, the conclusions that are derived from these techniques usually require additional care, since they can be significantly more ambiguous than deuterium NMR.

### 3.2. Dielectric Spectroscopy

Dielectric spectroscopy can provide insight into the dipole moment reorientation dynamics within solids [54]. Typically, the dependence of the complex permittivity (Equation (2)) of a material with respect to frequency and temperature is studied by building a parallel plate capacitor with the dielectric material of interest (i.e., the MOF) enclosed. The capacitance is measured—with respect to a reference capacitor—along a wide range of electric field frequencies and temperatures. The measured capacitance can be readily converted to dielectric permittivity when the dimensions of the capacitor are known.

(2) $$\varepsilon^{*}\left(\omega ,T\right)=\varepsilon^{\prime }\left(\omega ,T\right)-i\varepsilon^{\prime \prime }\left(\omega ,T\right)$$

The most common analysis in MOF literature involves identifying the maxima of the imaginary part of the complex permittivity—also known as the dielectric loss (ε″)—with respect to temperature or frequency. These are indicative of dipolar reorientations (also known as relaxations) in response to the applied alternating electric field [54] (pp. 59–61). Since this type of motion is thermally activated, a characteristic temperature shift in the relaxation peaks is usually observed, with higher frequencies corresponding to higher temperatures. A linear relation between the frequency of maximal ε’’ and the inverse of temperature is often found, from which an activation energy and a pre-exponential factor are obtained, according to the Arrhenius equation:

(3) $$\omega_{p}=\omega_{0}e^{\frac{-E_{a}}{kT}},$$

where ω_p_ is the frequency corresponding to the peak in ε″, ω_0_ is the pre-exponential factor (or attempt frequency), *E*_a_ is the activation energy, k is the Boltzmann constant, and *T* is the temperature at which the ω_p_ value is obtained. k*T*

In the field of MOFs, dielectric spectroscopy has been used mainly to study rotational motions of functionalized terephthalate linkers. Winston et al. [55] compared the response of IRMOF-1 (MOF-5) and IRMOF-2. Although they did not observe dielectric relaxations in the non-polar IRMOF-1, they did identify it in the bromine-substituted analog, and the signal could thus be produced by rotational motion of the permanent dipole present in the bromo-*p*-phenylene moiety. Fitting the spectra to an appropriate model led to an activation energy of 30.5 kJ mol^−1^. 

This is the case with some functionalized UiO-66(Zr) frameworks studied by Devautour et al. [56], where the dipole moments generated by the presence of Br and NH_2_ functionalities on the rings of the terephthalate linkers produced noticeable dielectric relaxation processes visible in the dielectric spectra. 

Frunza et al. [57] demonstrated the use of dielectric spectroscopy to characterize rotational motions in MOF-5. Their work suggests that, even in a non-polar framework, deformations in the structure possibly lead to the appearance of dipole moments related to linker rotation. The authors assigned a low activation energy process to bending of the SBUs of MOF-5, and a higher energy process (33.4–37.3 kJ mol^−1^) to phenylene rotational motions.

Dielectric spectroscopy has recently been used in other non-terephthalate-based frameworks. Knebel et al. [11] included this technique in their study of the rotational mobility of the 2-methylimidazolate linker in ZIF-8. Using a similar analysis to those in the previously mentioned literature, they found a dielectric relaxation corresponding to an activation energy of 49 kJ mol^−1^. They assigned this motion to the slow linker swinging that could also be observed in ^2^H NMR, albeit with a slightly higher activation energy obtained with the latter technique. 

Balčiūnas et al. [58] reported a comprehensive dielectric study of linker dynamics in ZIF-90—an isoreticular analog of ZIF-8 that has a carboxaldehyde group in place of the methyl group. Their work shows the effect that different gas molecules present in the pores have on the linker’s ability to perform partial rotations. The linker rotation in the evacuated ZIF-90 has an activation energy of 22 kJ mol^−1^, which increases to 34.7 and 35.7 kJ mol^−1^ in the presence of N_2_ and CO_2_, respectively.

It is important to note that dielectric spectroscopy cannot provide the same level of detail as deuterium NMR experiments regarding the nature and trajectory of molecular motions. As observed in the cited articles, no specific conclusions are made as to whether the rotators perform full 360° rotations, or their rotational amplitude in the case of the partial rotations. Nevertheless, dielectric spectroscopy is clearly growing in popularity in the MOF field as it is a powerful and sensitive method to probe dynamics of MOFs with the added benefit of not requiring chemical labelling.

### 3.3. Terahertz Spectroscopy

Very recently, far-infrared (FIR) spectroscopy in the terahertz range has been applied successfully to study MOF linker dynamics. This vibrational spectroscopy technique is used to study low-energy vibrational modes in solid materials, which tend to be related to structural flexibility [59]. As discussed in Section 2, librations are a prevalent form of partial rotational motion in MOF linkers, whose frequencies have been observed in the THz range. This is indeed the range where you expect rotational motions about a minimum in a potential energy. Dynamics of Type A—i.e., involving hopping over an energetic barrier—are thus not detectable by this method. 

High-quality quantum chemical calculations are usually required in order to interpret the spectra associated to THz spectroscopy. In such a manner, the vibrational modes belonging to linker rotation can be isolated from the various other types of collective modes.

**Figure 18:**
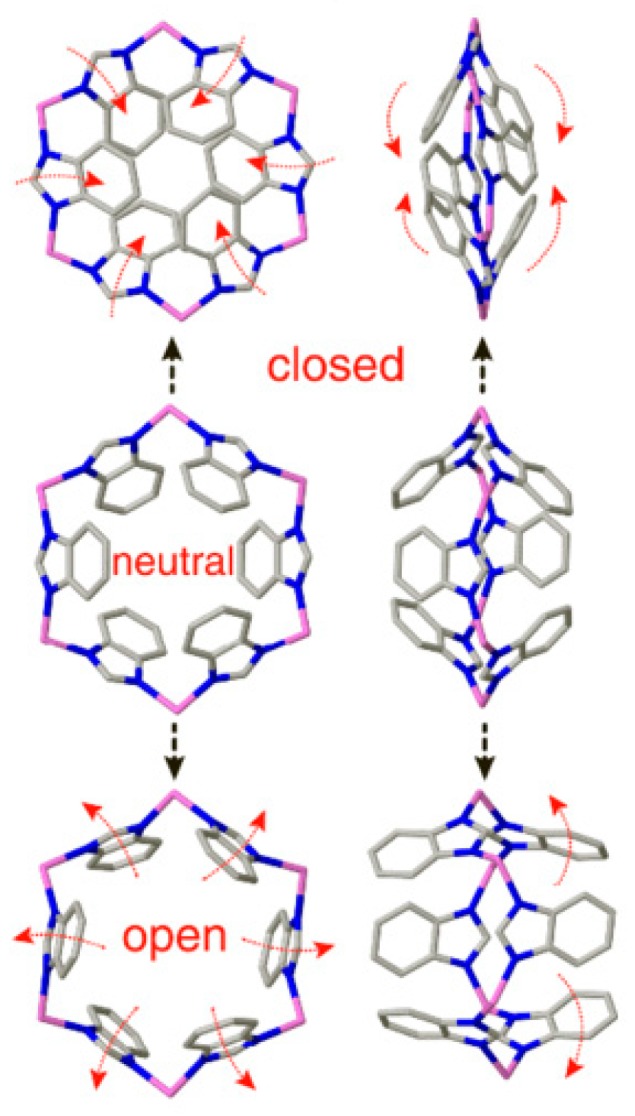
Illustration of low-energy vibration mode in ZIF-7, causing pore opening and closing. Reproduced with permission from [60]. Copyright American Physical Society, 2014.

Ryder et al. [60] undertook a detailed study of lattice dynamics in three ZIF materials (ZIF-4, ZIF-7, and ZIF-8) using far-infrared spectroscopy. Specifically, they focused on the frequency region below 21 THz, down to 0.6 THz, using synchrotron radiation. Although their report identifies a large array of low-frequency lattice vibrations, the relevant modes for linker rotation were found close to the lower limit of the probed range. Gate-opening modes were found in ZIF-4, ZIF-7, and ZIF-8 at 1, 1.47 and 1 THz, respectively. In all three cases, the modes involve librations of at least some of the imidazolate linkers that form one of the windows of the pore (example of ZIF-7 shown in Figure 18). In a similar manner, but using terahertz time-domain spectroscopy (THz-TDS), Tan et al. [61] identified vibrational modes involving rotational motion of the linkers in ZIF-8 and ZIF-90 in the same frequency range.

In a subsequent article, Ryder et al. [24] applied THz spectroscopy to identify the differences in the librational rotation of two types of terephthalate linkers—one significantly more hindered than the other—that form the MIL-140A framework (addressed in Section 2.2).

Additionally, Li et al. [39] used THz-TDS to detect the rotation of the methyl group in ZIF-8—one of the few examples of Type C rotors found in MOF literature.

Terahertz spectroscopy is likely at the start of its application in the field of linker rotation, yet the work discussed here demonstrates its great potential, when coupled with appropriate computational chemistry methods, to expand our understanding of partial rotational dynamics in MOFs.

### 3.4. Computational Methodologies

**Figure 19:**
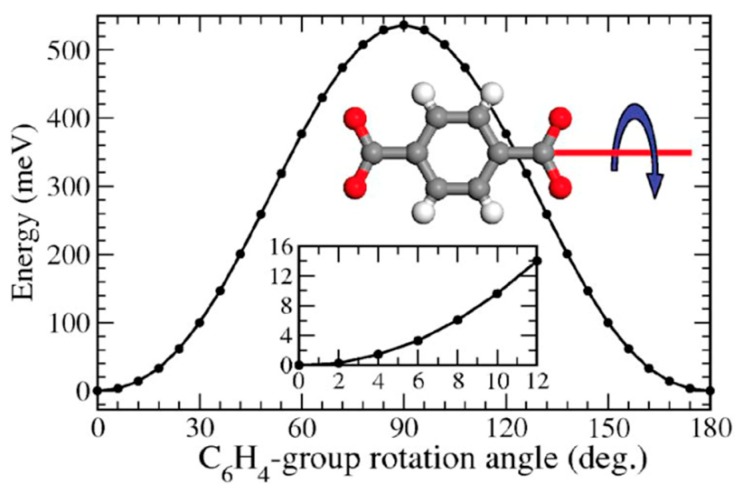
Energy profile for the rotation of a single *para*-phenylene unit in MOF-5 using density functional theory (DFT) single-point calculations. Reproduced with permission from [17]. Copyright American Physical Society, 2006.

The seminal works of Zhou et al. in 2006 [17], Kuc et al. in 2007 [62], and Winston et al. in 2008 [55] were the first to address the issue of linker rotation in metal–organic frameworks from a computational perspective. Zhou et al. studied the rotation of the *para*-phenylene units in MOF-5 using density functional theory (DFT) single-point calculations (see Figure 19). Their method involved the rotation of only one linker out of the six present in the primitive unit cell, with two-degree steps, from 0° to 180°. The rotational barrier found at 90° is 51.8 kJ mol^−1^. Kuc et al. studied the properties of over 30 MOF structures, at the semi-empirical level with Density functional based tight-binding (DFTB) calculations. As part of this larger study, they calculated a rotation barrier for the organic linker in IRMOF-1 (= MOF-5) of 34 kJ mol^−1^, a value significantly lower than that calculated by Zhou, and which can be attributed to the lower-accuracy level of theory chosen. Kuc et al. also stated that they observed “nearly free rotation of the linker” at 300 K using DFTB-based molecular dynamics, which is not in agreement with the later experimental findings through ^2^H NMR showing that at 300 K the flipping rate is <1 kHz [16].

Winston’s study focused more specifically on the rotation dynamics of the bromo-*p*-phenylene organic linkers— called “dipolar molecular rotors”—in IRMOF-2, by combining experimental and computational methods. In particular, the authors calculated rotational energy barriers by a series of energy calculations with 10° angle increments, at two levels of theory: density functional theory (DFT) using the B3LYP functional, and the post-Hartree–Fock Møller–Plesset (MP2) theory. However, these studies were performed not on the periodic crystalline structures, but on model clusters composed of a single linker and two metal nodes. The rotation barrier ranges between 20 and 31 kJ mol^−1^ depending on the computational method, which is in good agreement with the experimental value of 30.5 kJ mol^−1^ determined by the same authors using dielectric spectroscopy.

This methodology is the simplest way to computationally characterize the rotation of a given linker. The computational cost is relatively low, because it corresponds to a series of single-point energy calculations with rotated configurations of the linker (in some studies, the other coordinates are relaxed, but not always). This has been used in several other studies, and can be performed with periodic DFT to take into account the full crystal structure, as has been done for example by Kolokolov et al. [63] in their comparison of the dynamics of MIL-53(Cr) and MIL-47(V), two materials with the same diamond-shaped micropores. They used periodic DFT calculations to study the nature of the low-frequency vibration modes of the two materials, identifying the linker rotation modes as well as the corresponding rotational barriers—in agreement with ^2^H NMR experimental data. The methodology can also be extended to a full calculation of the rotation energy profile, calculating the potential energy surface *E*(*ɸ*) by rotating a linker by an angle *ɸ*—usually in series of calculations constraining the dihedral angle *ɸ*. Shustova et al. [25] studied in this way the phenyl ring dynamics in a tetraphenylethylene-bridged MOF. In another recent example, Hobday et al. [37] looked at the tuning of the swing motion of various functionalized imidazolate linkers in ZIFs. Ryder et al. [24] determined the rotational energy profiles for various MIL–140A vibration modes, using DFT calculations, to show that experimentally observed low-energy vibrations in THz spectroscopy originate from the hindered rotations of organic linkers.

However, the rotation of MOF linkers also involves entropic contributions—which are not taken into account in the potential energy surface scans—and can be expected to be significant, given that the soft vibration motions often have low frequency. These effects can be studied with molecular dynamics (MD) simulations at finite temperature *T*, where the free dynamics of the ligand will be sampled over time. Burtch et al. [64] investigated the framework dynamics of pillared zinc-based MOFs, relying on a classical molecular dynamics approach where an empirical force field is used to describe the various intra- and intermolecular terms of the energy. They also used this approach to study the influence of framework dynamics on water adsorption in these materials, and showed that the dynamics of the linker (DABCO, 1,4-diazabicyclo[2.2.2]octane) is strongly impacted by the presence of water guest molecules. However, the impact of the flexibility itself on total water uptake was found to be negligible [65].

In a recent paper, Namsani and Yazayin [66] performed MD simulations of halogen-functionalized IRMOF-1 and IRMOF-7 under the influence of an external electric field. Their study shows that the rotation angle of dipolar linkers may be controlled externally to enhance the diffusion of methane molecules in a specific direction, depending on the orientation of the electrical field.

We note here that several other studies have studied the impact of linker rotation on properties such as adsorption of gas or liquid molecules. This can be done either with a full description of the material’s flexibility (through MD simulations), or even at a lower level by a cursory look at the geometric impact of the rotation of the linker on the properties—without a full description of the energetics of the rotation itself. The Sholl group has published several studies on the impact of flexibility in ZIF-8 on adsorption, for example of methane, carbon dioxide [67], and noble gases [68]. Elsaidi et al. [69] described the impact of ring rotation upon gas adsorption in the nanoporous spaces of SIFSIX-3 pillared square grid networks.

**Figure 20:**
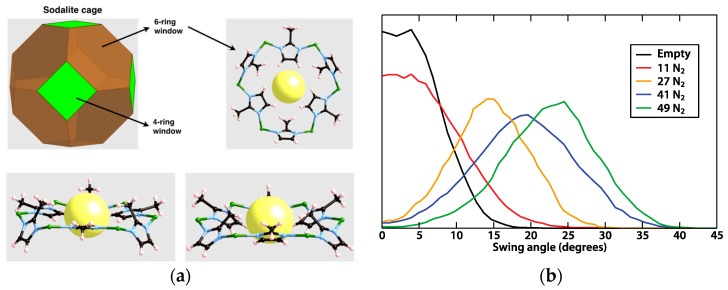
(**a**) Depiction of the “out of plane” swinging motion of the methylimidazolate linkers in the 6-ring window of ZIF-8 material, with sodalite topology; (**b**) distribution of swing angle due to thermal motion at 300 K, in the evacuated material and in presence of N_2_ adsorbate molecules. Reproduced with permission from [35]. Copyright Wiley-VCH, 2017.

While classical MD has a relatively low computational cost which allows the sampling of large time and length scales, its accuracy is limited by that of the intra- and intermolecular interaction potentials of its underlying force field. This accuracy can be particularly limited for soft vibration modes—which are of critical importance in soft porous crystals—and large dihedral rotation angles, as Burtch showed in their study [64]. Therefore, there is a need for molecular dynamics simulations based on quantum chemistry calculations of the interatomic forces, a method called ab initio molecular dynamics (AIMD). Recent studies have shown that the use of AIMD can shed light into the linker dynamics in MOFs, investigating the nature of the large-amplitude “swinging” motion of the imidazolate linkers that give rise to the flexibility of ZIF-8 (depicted in Figure 20a). This showed in particular that the deformation upon adsorption is continuous upon pore loading with nitrogen (see Figure 20b) [35], and highlighted the influence of the linker functionalization and the impact of this delicate balance of microscopic interactions on the framework’s overall flexibility [38].

Finally, direct sampling of the organic linker rotation through MD is suitable for low-barrier rotations that occur spontaneously within the timescale of MD simulations (from ps to ns). It will not be suitable to describe activated rotation events corresponding to high barriers, where free energy methods such as metadynamics will be necessary to sample the free energy profile *F*(*ɸ*) [70]. Such methods have been used extensively in the literature on the conformations of organic molecules and biological systems [71], but their use in the MOF field has been so far relatively rare. Haigis et al. [72] used metadynamics in conjunction with AIMD to probe the water-assisted breaking of the linker–metal bond that occurs during hydrothermal breakdown of MIL-53(Ga).

## 4. Implications of Rotational Dynamics on Applications of MOFs

### 4.1. Diffusion and Adsorption

There are ample studies where guest adsorption leads to a rotational conformational change [59,73,74,75,76]. These studies do not directly investigate the dynamics of linker rotation, but the aforementioned observation implies that linker rotation is energetically possible. As discussed in Section 2, the presence of guest molecules inside the pores generally decreases the rate of rotational dynamics of the linkers. A clear example of this is reported by Bracco et al. [29] (first discussed in Section 2.1, linker structure shown in Scheme 2, bottom) where the 180° flip motion of the 1,4-bis(1H-pyrazol-4-ylethynyl)benzene linker coordinated to Zn^2+^ decreases from 10^11^ Hz to 10^5^ Hz, along with an increase in the activation energy from 2 to 19 kJ mol^−1^ upon complete pore filling with CO_2_ at 150 K. At partial filling of CO_2,_ intermittent rotational frequencies due to a combination of static and mobile linker were found. Interestingly, when CO_2_ guest molecules are present, the librational motion alongside the flips has a larger amplitude (± 32–41°) with respect to the libration of the guest-free structure (amplitude of ±20°). This is an example of the influence of guest molecules of the rotational dynamics. Yet, the question we pose here is: what is the influence of linker rotational dynamics on guest diffusion and adsorption?

An often addressed phenomenon that arises from the interaction between a mobile linker and the guest molecules is the so-called gate-opening effect. It is usually observed as an inflection point, a step, or hysteresis in an adsorption isotherm, as a result of a structural transition in the MOF [77,78,79]. This structural transition can be due for example to the breathing effect [80]. Here we discuss the gate-opening effect with regard to structural transitions with a change in rotational conformation of the linkers.

**Figure 21:**
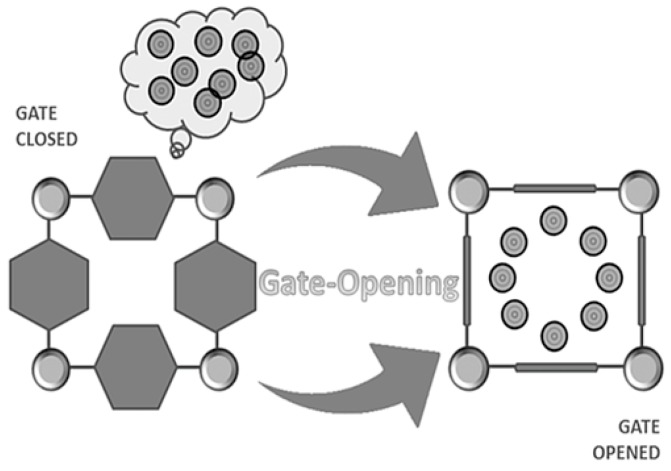
Illustration of the gate-opening effect. Reproduced with permission from [81]. Copyright Wiley-VCH, 2016.

The gate-opening can affect guest diffusion. Often the pore size expected based from XRD does not correspond with the size of the molecules that can be adsorbed: molecules larger than expected are able to diffuse into the pores. A typical example is ZIF-8. This is due to the librational movement of the imidazolate linker (see Section 2.2). This means that energetically the pore opening can easily be enlarged due to the librational motion of the linker. This is shown schematically in Figure 21.

More specifically, the 6-membered rings that provide entrance to the cages have a free diameter of ca. 3.0 Å. Many molecules with a kinetic diameter larger than that can penetrate into the pores, e.g. O_2_, N_2_, CH_4_ [82]. This was first described by Moggach et al. [4], in a study where ZIF-8 crystals were submitted to a pressure up to 1.47 GPa via a 4:1 (methanol:ethanol) hydrostatic mixture. This resulted in a single-crystal to single-crystal phase transition to a previously unobserved phase. This phase transition involves the rotation of the methylimidazolate linker, which leads to an increase in the diameter of the 6-membered rings from 3.0 to 3.6 Å, allowing more methanol and ethanol molecules to enter the pores. Later Fairen-Jimenez et al. [5] showed that this same phase transition also takes place upon gas adsorption at lower pressures. For example, for N_2_ at 77 K it occurs at ∼0.02 bar [83]. 

Recently, Hobday et al. [37] compared ZIF-8 and its carboxaldehyde and nitro functionalized analogues (ZIF-90 and ZIF-65, respectively) also under high hydrostatic pressure of a 4:1 methanol:ethanol mixture. At high pressures, the linker in ZIF-90 undergoes a similar rotation as in ZIF-8, which leads to a larger pore opening. In contrast, the linker in ZIF-65 rotates in the opposite direction, leading to a smaller pore opening. This suggests a difference in interaction energies of the –NO_2_ groups between each other and with the alcohol molecules, with respect to what occurs with the –CH_3_ and –CHO groups. The rotational dynamics of the nitro-functionalized ligand might still be different for other guest molecules.

For a framework constructed from Mn^2+^ and isonicotinate, the one-dimensional pores are lined with the isonicotinate linkers, which, in the guest-free conformation of the MOF, are orientated into a closed pore configuration that is too narrow for diffusion of small molecules such as CO_2_. However, adsorption of guest molecules such as CO_2_ and propane is associated with rotation of these linkers about their coordination axis into an open pore configuration. This leads to a steep rise in the adsorption curve at the gate-opening pressure. The gate-opening pressure is dependent on the adsorption energy and hence adsorbate dependent [81].

**Figure 22:**
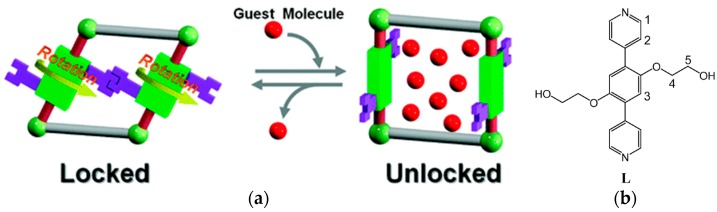
(**a**) Illustration of gate-opening effect in 2-D sheet framework with glycol functionalization; (**b**) Schematic structure of linker. Reproduced with permission from [84]. Copyright American Chemical Society, 2009.

An intriguing example of a material that is capable of transforming between a gate-closed to gate-open configuration via linker rotation was reported by Kitagawa et al. [84]. The structure is based on 2-D sheets that are pillared by a ligand with a 180° coordination geometry and an ethylene glycol functionalization (see Figure 22). These side chains act as a molecular gate, that can be unlocked by guest interactions. The hydrogen bonds of the ethylene glycol side chain are vital in the mechanism. In the guest free framework, the side chains form hydrogen bonds with each other that lead to an effectively closed pore configuration: N_2_ and O_2_ cannot be adsorbed. In contrast, water and methanol can be adsorbed and lead to a gate-opening of the pores. The hydrogen bonds between the ethylene glycol groups are broken as H-bonds with the guest molecules are formed. This leads to a rotation of the linker around its coordination axis leading to a large pore opening (see Figure 22). At the vapor pressure upon which this occurs the sorption isotherm shows a step that is typical of a gate-opening effect. For CO_2_, due to its much smaller tendency to form H-bonds with the ethylene glycol groups this happens at a much higher vapor pressure.

In a MOF based on Cu^2+^ and 5-diethyl-1,2,4-triazole, the ethyl groups are capable of momentarily moving out of the way to permit the diffusion of guest molecules that are normally too large to fit, such as acetylene and CO_2_ [85]. The structure of the framework is hardly changed upon guest inclusion. This is a clear example of linker molecules, or part thereof, only temporarily moving out of the way.

Zhang et al. [86] studied a framework based on Cu^2+^, which is pillared by hexafluorogermanate or hexafluoroniobate as well as a ligand containing two pyridyl rings, 4,4’-dipyridylacetylene or 4,4’-azopyridine more specifically. In the guest-free state one of the pyridyl rings is rotated such as to optimize a favorable C–H···F hydrogen bond. When solvent molecules are occluded, both pyridyl rings have a close to planar conformation, which leads to a larger pore size. The difference is the largest for the 4,4’-azopyridine ligand, with a difference of 30° in rotational conformation for one pyridyl ring in the guest-free state. The result is that the solvated MOF has larger pore sizes: 4.2 Å instead of 3 Å. This larger pore size of 4.2 Å should allow for butadiene to diffuse through, while the butene isomers that have a larger cross section cannot. Indeed, the authors report both plausible gate-opening in the adsorption isotherm of butadiene, and a good separation between butadiene and butene isomers. Often, MOFs do not perform as well in breakthrough adsorption experiments as expected from the single gas adsorption experiments [87,88,89]. However, in this case a very good selectivity was retained in the breakthrough experiments.

**Figure 23:**
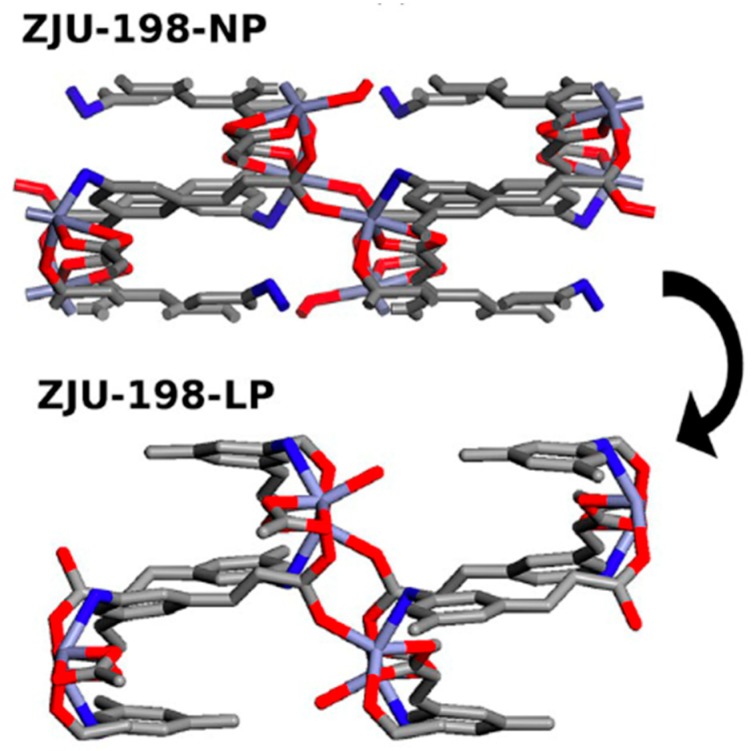
NP and LP conformations of ZJU-198 framework. Reproduced with permission from [90]. Copyright Wiley-VCH, 2018.

For ZJU-198, shown in Figure 23 as ZJU-198-NP (NP = narrow pore), it has been shown experimentally that this structure can separate CO_2_ from N_2_, acetylene from methane, and, moderately, acetylene from ethylene [91]. This was initially rationalized via the static ZJU-198-NP structure. Yet, many of the adsorbate molecules in fact do not fit into the ZJU-198-NP pores. Via Monte Carlo simulations through grand canonical assemblies and molecular dynamics, Calero et al. [90] showed that the structure actually undergoes a phase transition upon guest adsorption, via a linker rotation that leads to large pore cavities. This structure is shown as ZJU-198-LP (LP = large pore) in Figure 23. This breathing effect leads to preferential adsorption of molecules that favor the large-pore configuration. In fact, the authors could far better reproduce the experimental adsorption isotherms and selectivity, including this framework flexibility than by assuming a static ZJU-198-NP structure. 

So far, we discussed the rotation-induced gate-opening effect that leads to larger effective pore windows than predicted from XRD crystal structures. This can happen if the linkers in the equilibrium guest-filled structure have a different orientational conformation with larger pore size. It can also happen due to transitory linker rotation that allows diffusion of guest molecules, while the equilibrium conformation is very alike that in the guest-free structure. The result of this effect is that molecules larger than expected from the refined guest-free structure can diffuse through the MOF.

Another way in which rotational dynamics can lead to different adsorbate/adsorbent interactions is when a conformational transition of the MOF affords a better packing of the adsorbate molecules, or when it leads to more favorable adsorbate/adsorbent interactions. 

The above-discussed gate-opening effect is in fact more intricate than initially thought. A gradual deformation of the ZIF-8 structure takes place during adsorption. In the case of small molecules—such as N_2_, Ar, O_2_ or CO—at low temperature, this deformation can lead to stepped isotherms due to packing effects. More specifically, a step occurs when a more favorable packing arrangement of guest molecules becomes possible in the deformed structure. As such, the polarizability and molecular size and shape of the gases play a significant role in the adsorption behavior [35,92]. Recently Coudert et al. [38], via a combination of N_2_ sorption and first principles thermodynamics, showed that the isoreticular ZIF-8-Cl (ZIF-8 with the –CH_3_ group substituted by –Cl) has similar steps in the low temperature N_2_ adsorption. Here too, it is convincingly shown that this is governed by an increasing rotational linker deformation and guest packing effects. In contrast, the isoreticular ZIF-8-Br was shown to be stiff and displays a characteristic type Ia adsorption isotherm. 

**Scheme 4:**
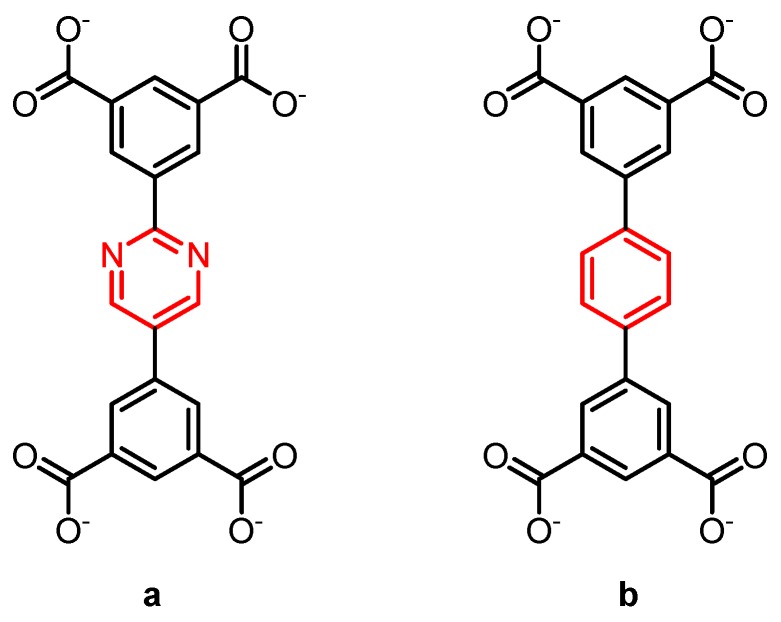
Two rotor linkers used in (**a**) UTSA-76 and (**b**) NOTT-101.

In two metal–organic framework based on Cu^2+^-paddle wheels, that contain the linkers shown in Scheme 4, a difference in rotational dynamics between these linkers leads to distinct differences in the adsorption of methane [27]. At high loading, the pyrimidine-containing linker has a much higher methane uptake. At low methane pressures, both frameworks show a very similar uptake. DFT calculations show similar adsorption energies for both MOFs. With quasi-elastic neutron scattering, the authors of this study found that the pyrimidine containing linker is far more dynamic, and hence the authors postulated that the central pyrimidine rings can be more easily adjusted and oriented to optimize the methane packing in the MOF at high pressure than that this can happen for the central benzene rings in the other MOF.

In another series of MOFs investigated for methane storage (discussed in Section 2.1, see Figure 8), a difference in rotational dynamics for the different ligands occurs. However, it is less clear in these structures what the precise effect of rotational dynamics is on the interplay of flexibility, pore shape, and adsorption energies that leads to the observed differences in methane loading.

SIFSIX-3-Ni consists of a network based on square grids of Ni^2+^ and pyrazine, which are pillared by SiF_6_^2−^. This structure shows a distinct inflection point in the Xe adsorption isotherm that is absent for Kr, N_2_, and CO_2_. The pore diameters based on the refined structure are 3.5–3.8 Å, which is close to the kinetic diameter of 4 Å of Xe. Whether the structure is present in vacuum, or fully loaded with He or Xe, the pyrazine rings within the framework have a similar orientation, where each ring is rotated approximately 16° with respect to the square grid plane orientation [69]. Two different orientations (−16° and 16°) are possible. The transition that occurs around the inflection point is hence subtler. Aided by DFT calculations, the authors concluded that a disorder-to-order structural transition in the organization of the pyrazine rings takes place, which leads to an organized pattern of aligned +16° and −16° pyrazine configurations that can better accommodate Xe guests molecules. In this case, it is not so much a packing effect, but rather a reorganization of the linker conformations to allow for optimal adsorbate/adsorbent interactions.

For an isoreticular structure based on Zn^2+^ and Ni^2+^ it has also been shown that the rotational dynamics of the pyrazine rings play a role in the adsorption of CO_2_ [93]. Here, intriguingly, a higher uptake of CO_2_ is observed at 298 K than at 195 K.

Gee and Sholl [94] applied molecular dynamics simulation to better grasp what the role of rotational flexibility of MIL-47 and MOF-48 might be on the adsorption selectivity for different pairs of C8 aromatics. These MOFs with one-dimensional pores are based on V^4+^ coordinated by oxygen and terephthalic acid (MIL-47) or dimethyl terephthalic acid (MOF-48). They compared static Grand Canonical Monte Carlo simulations with GCMC simulations on a series of snapshots taken from flexible molecular dynamics simulations that pertain to a wide set of dihedral angles of the terephthalate linkers. When rotational flexibility was included, the computational results matched the experimentally observed selectivity far better. For these host/guest systems, the simulated selectivity decreased when flexibility was included.

As is clear, the rotational dynamics of linker molecules in MOFs can have a profound effect on the adsorption properties of MOFs. The gate-opening effect shows that the effective pore size that should be considered to predict selectivity based on a molecular sieving effect, is in this case generally larger than the pore size determined from the guest-free refined structure. In addition, the potential for structural transitions based on rotational linker dynamics, can lead to a better packing of guest molecules at high loadings. This is especially the case for small molecules at low temperatures these packing effects may be pronounced.

The examples we discussed show that in fact rotational dynamics has the potential to increase or decrease the adsorption selectivity, depending on the specific interplay between the MOF structure, its flexibility and the guest molecules. In any case, in order to understand the adsorptive properties of MOFs, the rotational dynamics need to be considered. 

### 4.2. Optical Properties

**Figure 24:**
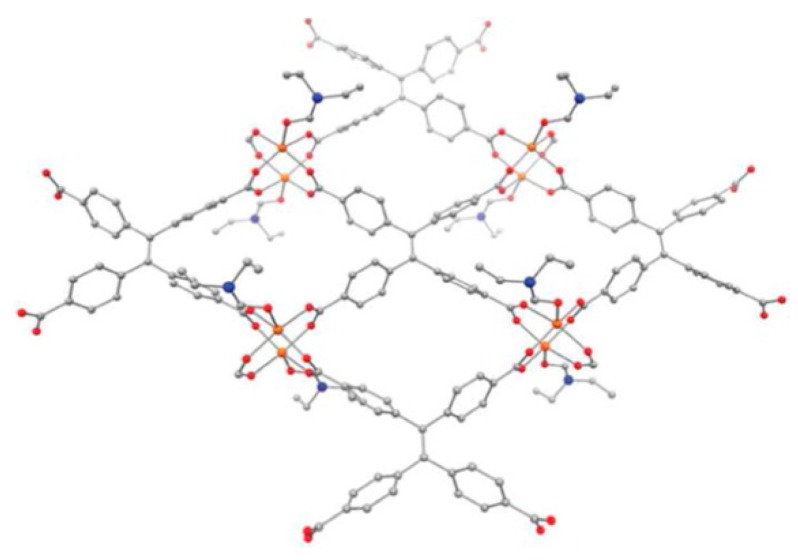
Crystal structure of the fluorescent Zn_2_(TCPE) framework. Reproduced with permission from [25]. Copyright American Chemical Society, 2012.

Rotational dynamics can influence several optical properties of MOFs. One area where this happens is inspired by the recently discovered phenomenon of aggregation-induced emission (AID) [95]. Chromophores that have this property are far more fluorescent as aggregates than when they are present in dilute solutions. In these chromophores, orbitals that are involved in the luminescence decay pathway are present on groups that are rapidly rotating in solution, hence quenching the fluorescence. When aggregated, this rotation is hindered, hence fluorescence is switched on [96]. A typical example is the molecule tetraphenylethylene (TPE), for which fast rotation of phenyl groups and twisting of the C=C group occur in solution, but not upon aggregation [97]. Dinca et al. [98] showed that a tight packing of the chromophores is actually not necessary, since simply restraining the mobility via incorporation the chromophore in a matrix can suffice for fluorescence to occur. More specifically, a tetrakis(4-carboxyphenyl)ethylene (TCPE) ligand was built as linker into metal–organic frameworks via either Zn^2+^ (see Figure 24) or Cd^2+^ ions. Despite the distance between the TPE motifs being significantly larger in the MOF than in the aggregate, and rotation/flipping of the phenyl rings being feasible, the MOFs exhibit fluorescence, both in the solvated and unsolvated state.

The same group investigated the effect for the MOF built up with Zn^2+^ paddle wheels in greater detail via ^2^H NMR and DFT modeling of the energy barrier for rotation on a cluster model. With respect to free TPE in the gas phase, the linker in the MOF has twice the energy barrier for ring rotation: 50 kJ/mol versus 25 kJ/mol. This is due to the geometrical constraint the MOF topology poses on the linker which leads to steric hindrance upon ring rotation that is absent from the free TPE [98]. Apparently, these decreased phenyl rotations suffice to retain the fluorescence of the materials. The authors predict that if the MOF topology could be chosen such that the rotational energy barrier could be lowered significantly, they could be interesting sensors. They postulate that in such cases the guest-free MOF should hardly fluoresce, while upon guest inclusion the fluorescence should be switched on [25]. 

Correspondingly, Du et al. [99] recently hypothesized that the fluorescence enhancement of MIL-53 related MOFs with Au particles attached in the presence of glutathione, is related to a restriction of the rotational dynamics of the terephthalate linkers.

Serra-Crespo et al. [100] showed that modulation of linker dynamics can also act as the switch to turn on the non-linear optical (NLO) response of NH_2_-MIL-53(Al). NH_2_-MIL-53 is capable of generating second-order NLO light, which is only allowed for non-centrosymmetric structures, in the closed pore configuration where the linkers experience significant steric hindrance to rotation. When guest molecules were introduced, the structure transformed to an open pore configuration that allowed for linker rotation. This, in turn, lead to a randomization of the linker orientation that switched off the NLO response.

In short, linker dynamics can have a pronounced effect on optical properties such as fluorescence and non-linear optical light generation, such that on–off transitions may be realized based on the drastic changes in linker dynamics upon guest adsorption/desorption. 

### 4.3. Mechanical Properties

Metal–organic frameworks can show intriguing mechanical properties, such as negative thermal expansion or gigantic positive thermal expansion [101,102,103,104,105,106]. This prompts the question: in what ways does the flexibility that many MOFs have play a role in these phenomena? Within the frame of this review, what role may rotational linker dynamics play? A few papers have addressed this explicitly.

For MOF-5, which shows negative thermal expansion between at least 80 K and 500 K, it has been shown in an experimental [103] and a computational study [106] that its unit cell contraction with increasing temperature is due to a concerted transverse movement of the linkers and the twisting and vibrations of the carboxylate groups specifically. Yet, the librations of the terephthalate linkers hardly play a role in the phenomenon.

**Figure 25:**
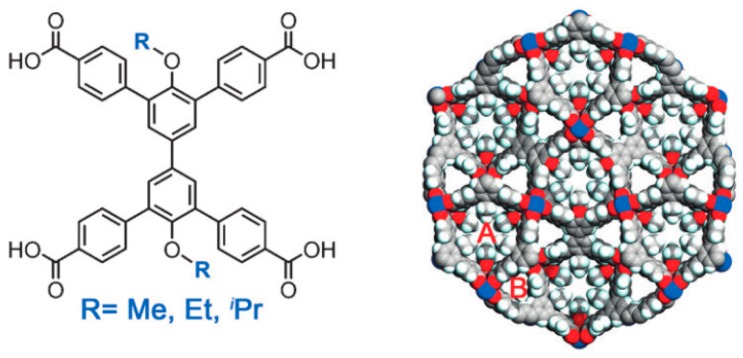
Structure of the FJI-H11-R framework and its rotor linker. Adapted with permission from [107]. Copyright Wiley-VCH, 2016.

The FJI-H11-R MOFs—which consist of Cu_2_ paddle wheels and the rather elaborate linkers depicted in Figure 25—show a significant positive thermal expansion [107], with a slight contraction of 0.6 % of the a-axis between 100 K and 293 K, but a large positive thermal expansion of 9.4 % along the c-axis, leading to a pore volume increase of 8.0 %. The authors showed that this is related to rotation of the of *p*-phenylene groups in the linker, and deformation of the linker.

## 5. Conclusions

In this review we categorized rotational linker dynamics in MOFs into four types (A–D), based on geometric and energetic considerations. Type A occurs in linkers with straight axles and low enough rotational barriers, such that they are capable of completing 360° rotations. Type B consists of partial rotations, such as librations (oscillations about a minimum) and hops in energetically constrained rotors. Types C and D are topologically different from the first two, and far fewer examples are reported in literature. In Type C, the rotor is a side functional group that is not involved in the connectivity, while in Type D the rotor is formed by a macrocyclic molecule that is mechanically interlocked around the main linker strut. In general, rotational motions are commonplace in MOFs, with a rich diversity of mechanisms of rotation, as well as complex interplays between the rotor framework and guest molecules.

Given the diverse examples found in the literature, it is evident that electronic configuration of the rotor and the steric environment inside the framework are the two main factors determining the torsional potential. Hence, they are essential parameters in the design of framework rotor systems. For instance, high rotational barriers can be obtained using linkers that feature π-electron delocalization systems. By contrast, axles with pure single-bond character—or with triple bond moieties—should be chosen in order to reach free rotational motion. Additionally, the coordination geometry of a linker may exclude the possibility of 360° rotations in certain types of MOFs. Steric hindrance on the rotors, arising from the framework itself or from guest molecules in the pores, will lead to increased rotational barriers.

With regard to the set of tools that can be used to study rotational dynamics of linkers, ^2^H NMR is evidently the most important in the field. This is due to its specificity and ease of interpretation. It has been used successfully to determine rotation steps, rates, and barriers of numerous and often diverse MOFs. Other methods, such as ^1^H NMR spin-lattice relaxation, dielectric spectroscopy, and THz spectroscopy, are becoming more commonplace, but may need further development and/or combination with supporting techniques to avoid ambiguity. Molecular modeling techniques can also be used to provide information on the dynamic nature of the frameworks. Relatively low-cost DFT calculations can be used to characterize the low-energy vibration modes of the materials, as well as the energy profile for linker rotation. More advanced methods, including ab initio molecular dynamics and free energy methods (such as metadynamics), are starting to appear in the study of rotational linker dynamics in MOFs, although their computational cost is significantly higher.

In addition to their prevalence in MOFs, these motions can have a drastic impact on their properties and on their performance in several applications. With regard to adsorption and separation, molecules larger than the pore size expected from the XRD refined structure might adsorb. Furthermore, the rotational dynamics allow the frameworks to access different conformational structures that allow better packing and/or more favorable adsorbate/adsorbent interactions for specific molecules. As such, the adsorption selectivity and capacity can be heavily influenced by the rotational dynamics. Still, the majority of adsorption simulations in MOFs treat the adsorbing materials as rigid, which may not capture the actual behavior of the MOF. As the rotational dynamics of the linkers are generally restricted by guest inclusion, it provides a pathway for reversible guest-dependent optical properties, such as switching on of fluorescence and switching-off second-harmonic generation upon guest inclusion.

Even though rotational dynamics are fairly prevalent in MOFs, their impact is often understated or entirely ignored. Traditional microporous materials, such as zeolites and activated carbon cannot display similar rotational dynamics. Yet, other emerging families of microporous materials, like porous organic frameworks (POFs) and porous organic cages should be expected to exhibit similar behavior. So far, research has primarily analyzed the properties that derive from rotation, but we expect growth in the exploitation of rotation as a valuable design factor in functional nanomaterials. In particular, more explicit cases of modulation of gas adsorption based on tunable rotational dynamics can be forecast, as the interplay between these two phenomena is becoming more understood. An especially exciting prospect is the attainment of coherent motion within MOFs, which might enable these ordered microporous materials to behave as artificial nanomachine platforms able to perform useful work.

## Figures and Tables

**Table 1:** Type A rotor linkers and their rotational barriers.

MOF	Rotator	Type	*T*/K*	Barrier/kJ mol^−1^	ω_0_/Hz	Method	Ref.
**MOF-5**	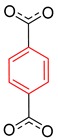	π-flip	363–435	47.3 ± 8.4	1.60 × 10^12^	^2^H NMR	[16]
**MOF-5**		π-flip	-	51.8	-	DFT	[17]
**MIL-53(Cr)**		π-flip	333–453	41	1.26 × 10^11^	^2^H NMR	[18]
**MIL-47(V)**		π-flip	373–483	45	8.80 × 10^10^	^2^H NMR	[18]
**MIL-53(Al)**		π-flip	359–492	37 ± 1	0.6 × 10^10^	^2^H NMR	[19]
**UiO-66(Zr)**		π-flip	213–403	30 ± 2	0.5 ± 0.8 × 10^12^	^2^H NMR	[22]
**MIL-140**		π-flip	-	27.4	-	DFT	[24]
**DMOF**		π-flip	253–359	32–36	0.3–10 × 10^11^	^2^H NMR	[23]
**MFM-180**	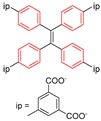	4-site	223–503	26; 28	1.6 × 10^11^; 3 × 10^7^	^2^H NMR	[9]
**MFM-181**	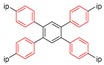	4-site	153–473	20; 34	9 × 10^11^; 4.6 × 10^8^	^2^H NMR	[9]
**BODCA-MOF**	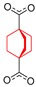	6-site; 3-site; free	6–12; 12–50; 50–292	0.8	4.7 × 10^10^	^1^H T_1_ relaxation	[10]
**MFM-112a**	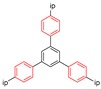	4-site	123–203	8.6	3 × 10^8^; 18 × 10^8^	^2^H NMR	[12]
**MFM-115a**	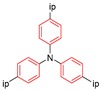	4-site; 6-site	203–315	14; 40	2 × 10^8^; 5 × 10^12^	^2^H NMR	[12]
**Zn_2_(TCPE)(DEF)_2_**	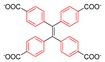	π-flip	300–421	43(6)	2.2 × 10^11^	^2^H NMR	[25]
**CdNa(2-stp)(pyz)_0.5_(H_2_O)**	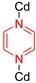	4-site	193–293	7.7	2.40 × 10^6^	^2^H NMR	[26]
**Zn_2_(1,4-ndc)_2_(DABCO)**	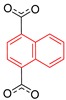	4-site	193–293	53	Not reported	^2^H NMR	[26]
**UTSA-76**	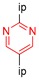	π-flip	-	8.2	-	DFT	[27]
**NOTT-101a**	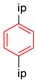	π-flip	-	20.2	-	DFT	[27]
**UCLA-R3**	Figure 9	3-site	-	56.5	8.7 × 10^15^	^2^H NMR	-
**Cu(bbcbco)(H_2_O)**	Scheme 2	-	-	15.5	-	DFT	[28]
**Zn-BPEB**	Scheme 2	free	150–293	2	2.2 × 10^12^	^1^H T_1_ relaxation	[29]

* Temperature range in which the experiments were performed.

## References

[1] Furukawa H., Cordova K.E., O’Keeffe M., Yaghi O.M. (2013). The Chemistry and Applications of Metal-Organic Frameworks. Science.

[2] Schneemann A., Bon V., Schwedler I., Senkovska I., Kaskel S., Fischer R.A. (2014). Flexible Metal–organic Frameworks. Chem. Soc. Rev..

[3] Khuong T.-A.A.V., Nunez J.E., Godinez C.E., Garcia-Garibay M.A., Nuñez J.E., Godinez C.E., Garcia-Garibay M.A. (2006). Crystalline Molecular Machines: A Quest toward Solid-State Dynamics and Function. Acc. Chem. Res..

[4] Moggach S.A., Bennett T.D., Cheetham A.K. (2009). The Effect of Pressure on ZIF-8: Increasing Pore Size with Pressure and the Formation of a High-Pressure Phase at 1.47 GPa. Angew. Chemie Int. Ed..

[5] Fairen-Jimenez D., Moggach S.A., Wharmby M.T., Wright P.A., Parsons S., Düren T. (2011). Opening the Gate: Framework Flexibility in ZIF-8 Explored by Experiments and Simulations. J. Am. Chem. Soc..

[6] Catalano L., Naumov P. (2018). Exploiting Rotational Motion in Molecular Crystals. CrystEngComm.

[7] Vogelsberg C.S., Garcia-Garibay M.A. (2012). Crystalline Molecular Machines: Function, Phase Order, Dimensionality, and Composition. Chem. Soc. Rev..

[8] Khudozhitkov A.E., Kolokolov D.I., Stepanov A.G. (2018). Characterization of Fast Restricted Librations of Terephthalate Linkers in MOF UiO-66(Zr) by ^2^H NMR Spin–Lattice Relaxation Analysis. J. Phys. Chem. C.

[9] Moreau F., Kolokolov D.I., Stepanov A.G., Easun T.L., Dailly A., Lewis W., Blake A.J., Nowell H., Lennox M.J., Besley E. (2017). Tailoring Porosity and Rotational Dynamics in a Series of Octacarboxylate Metal-Organic Frameworks. Proc. Natl. Acad. Sci. USA.

[10] Vogelsberg C.S., Uribe-Romo F.J., Lipton A.S., Yang S., Houk K.N., Brown S., Garcia-Garibay M.A. (2017). Ultrafast Rotation in an Amphidynamic Crystalline Metal Organic Framework. Proc. Natl. Acad. Sci. USA.

[11] Knebel A., Geppert B., Volgmann K., Kolokolov D.I., Stepanov A.G., Twiefel J., Heitjans P., Volkmer D., Caro J. (2017). Defibrillation of Soft Porous Metal-Organic Frameworks with Electric Fields. Science.

[12] Yan Y., Kolokolov D.I., da Silva I., Stepanov A.G., Blake A.J., Dailly A., Manuel P., Tang C.C., Yang S., Schröder M. (2017). Porous Metal–Organic Polyhedral Frameworks with Optimal Molecular Dynamics and Pore Geometry for Methane Storage. J. Am. Chem. Soc..

[13] Kottas G.S., Clarke L.I., Horinek D., Michl J. (2005). Artificial Molecular Rotors. Chem. Rev..

[14] Gonzalez J., Nandini Devi R., Tunstall D.P., Cox P.A., Wright P.A. (2005). Deuterium NMR Studies of Framework and Guest Mobility in the Metal–organic Framework Compound MOF-5, Zn_4_O(O_2_CC_6_H_4_CO_2_)_3_. Microporous Mesoporous Mater..

[15] Spiess H.W. (1983). Molecular Dynamics of Solid Polymers as Revealed by Deuteron NMR. Colloid Polym. Sci..

[16] Gould S.L., Tranchemontagne D., Yaghi O.M., Garcia-Garibay M.A. (2008). Amphidynamic Character of Crystalline MOF-5: Rotational Dynamics of Terephthalate Phenylenes in a Free-Volume, Sterically Unhindered Environment. J. Am. Chem. Soc..

[17] Zhou W., Yildirim T. (2006). Lattice Dynamics of Metal-Organic Frameworks: Neutron Inelastic Scattering and First-Principles Calculations. Phys. Rev. B.

[18] Kolokolov D.I., Jobic H., Stepanov A.G., Guillerm V., Devic T., Serre C., Férey G. (2010). Dynamics of Benzene Rings in MIL-53(Cr) and MIL-47(V) Frameworks Studied by ^2^H NMR Spectroscopy. Angew. Chem. Int. Ed..

[19] Kolokolov D.I., Stepanov A.G., Jobic H. (2014). Guest Controlled Rotational Dynamics of Terephthalate Phenylenes in Metal-Organic Framework MIL-53(Al): Effect of Different Xylene Loadings. J. Phys. Chem. C.

[20] Khudozhitkov A.E., Jobic H., Freude D., Haase J., Kolokolov D.I., Stepanov A.G. (2016). Ultraslow Dynamics of a Framework Linker in MIL-53 (Al) as a Sensor for Different Isomers of Xylene. J. Phys. Chem. C.

[21] Khudozhitkov A.E., Jobic H., Kolokolov D.I., Freude D., Haase J., Stepanov A.G. (2017). Probing the Guest-Mediated Structural Mobility in the UiO-66(Zr) Framework by ^2^H NMR Spectroscopy. J. Phys. Chem. C.

[22] Kolokolov D.I., Stepanov A.G., Guillerm V., Serre C., Frick B., Jobic H. (2012). Probing the Dynamics of the Porous Zr Terephthalate UiO-66 Framework Using ^2^H NMR and Neutron Scattering. J. Phys. Chem. C.

[23] Khudozhitkov A.E., Kolokolov D.I., Stepanov A.G., Bolotov V.A., Dybtsev D.N. (2015). Metal-Cation-Independent Dynamics of Phenylene Ring in Microporous MOFs: A ^2^H Solid-State NMR Study. J. Phys. Chem. C.

[24] Ryder M.R., Van De Voorde B., Civalleri B., Bennett T.D., Mukhopadhyay S., Cinque G., Fernandez-Alonso F., De Vos D., Rudić S., Tan J.C. (2017). Detecting Molecular Rotational Dynamics Complementing the Low-Frequency Terahertz Vibrations in a Zirconium-Based Metal-Organic Framework. Phys. Rev. Lett..

[25] Shustova N.B., Ong T.-C., Cozzolino A.F., Michaelis V.K., Griffin R.G., Dincă M. (2012). Phenyl Ring Dynamics in a Tetraphenylethylene-Bridged Metal–Organic Framework: Implications for the Mechanism of Aggregation-Induced Emission. J. Am. Chem. Soc..

[26] Horike S., Matsuda R., Tanaka D., Matsubara S., Mizuno M., Endo K., Kitagawa S. (2006). Dynamic Motion of Building Blocks in Porous Coordination Polymers. Angew. Chem. Int. Ed..

[27] Li B., Wen H.-M., Wang H., Wu H., Tyagi M., Yildirim T., Zhou W., Chen B. (2014). A Porous Metal–Organic Framework with Dynamic Pyrimidine Groups Exhibiting Record High Methane Storage Working Capacity. J. Am. Chem. Soc..

[28] Bastien G., Lemouchi C., Wzietek P., Simonov S., Zorina L., Rodríguez-Fortea A., Canadell E., Batail P. (2014). A Crystalline Hybrid of Paddlewheel copper(II) Dimers and Molecular Rotors: Singlet-Triplet Dynamics Revealed by Variable-Temperature Proton Spin-Lattice Relaxation. Z. Anorg. Allg. Chem..

[29] Bracco S., Castiglioni F., Comotti A., Galli S., Negroni M., Maspero A., Sozzani P. (2017). Ultrafast Molecular Rotors and Their CO_2_ Tuning in MOFs with Rod-Like Ligands. Chem. A Eur. J..

[30] Pakhira S., Takayanagi M., Nagaoka M. (2015). Diverse Rotational Flexibility of Substituted Dicarboxylate Ligands in Functional Porous Coordination Polymers. J. Phys. Chem. C.

[31] Inukai M., Tamura M., Horike S., Higuchi M., Kitagawa S., Nakamura K. (2018). Storage of CO_2_ into Porous Coordination Polymer Controlled by Molecular Rotor Dynamics. Angew. Chemie Int. Ed..

[32] Inukai M., Fukushima T., Hijikata Y., Ogiwara N., Horike S., Kitagawa S. (2015). Control of Molecular Rotor Rotational Frequencies in Porous Coordination Polymers Using a Solid-Solution Approach. J. Am. Chem. Soc..

[33] Jiang X., Duan H.-B., Khan S.I., Garcia-Garibay M.A. (2016). Diffusion-Controlled Rotation of Triptycene in a Metal–Organic Framework (MOF) Sheds Light on the Viscosity of MOF-Confined Solvent. ACS Cent. Sci..

[34] Liu Y., Her J.H., Dailly A., Ramirez-Cuesta A.J., Neumann D.A., Brown C.M. (2008). Reversible Structural Transition in MIL-53 with Large Temperature Hysteresis. J. Am. Chem. Soc..

[35] Coudert F.X. (2017). Molecular Mechanism of Swing Effect in Zeolitic Imidazolate Framework ZIF-8: Continuous Deformation upon Adsorption. ChemPhysChem.

[36] Kolokolov D.I., Stepanov A.G., Jobic H. (2015). Mobility of the 2-Methylimidazolate Linkers in ZIF-8 Probed by ^2^H NMR: Saloon Doors for the Guests. J. Phys. Chem. C.

[37] Hobday C.L., Bennett T.D., Fairen-Jimenez D., Graham A.J., Morrison C.A., Allan D.R., Düren T., Moggach S.A. (2018). Tuning the Swing Effect by Chemical Functionalization of Zeolitic Imidazolate Frameworks. J. Am. Chem. Soc..

[38] Chaplais G., Fraux G., Paillaud J.-L., Marichal C., Nouali H., Fuchs A.H., Coudert F.-X., Patarin J. (2018). Impacts of the Imidazolate Linker Substitution (CH_3_, Cl or Br) on the Structural and Adsorptive Properties of ZIF-8. J. Phys. Chem. C.

[39] Li Q., Zaczek A.J., Korter T.M., Zeitler J.A., Ruggiero M.T. (2018). Methyl-Rotation Dynamics in Metal–organic Frameworks Probed with Terahertz Spectroscopy. Chem. Commun..

[40] Zhou W., Wu H., Udovic T.J., Rush J.J., Yildirim T. (2008). Quasi-Free Methyl Rotation in Zeolitic Imidazolate Framework-8. J. Phys. Chem. A.

[41] Zheng B., Fu F., Wang L.L., Yang L., Zhu Y., Du H. (2018). Investigation of the Linker Swing Motion in the Zeolitic Imidazolate Framework ZIF-90. J. Phys. Chem. C.

[42] Morris W., Taylor R.E., Dybowski C., Yaghi O.M., Garcia-Garibay M.A. (2011). Framework Mobility in the Metal-Organic Framework Crystal IRMOF-3: Evidence for Aromatic Ring and Amine Rotation. J. Mol. Struct..

[43] Loeb S.J. (2007). Rotaxanes as Ligands: From Molecules to Materials. Chem. Soc. Rev..

[44] Deng H., Olson M.A., Stoddart J.F., Yaghi O.M. (2010). Robust Dynamics. Nat. Chem..

[45] Vukotic V.N., Harris K.J., Zhu K., Schurko R.W., Loeb S.J. (2012). Metal–organic Frameworks with Dynamic Interlocked Components. Nat. Chem..

[46] Zhu K., Vukotic V.N., O’Keefe C.A., Schurko R.W., Loeb S.J., Okeefe C.A., Schurko R.W., Loeb S.J., O’Keefe C.A., Schurko R.W. (2014). Metal–Organic Frameworks with Mechanically Interlocked Pillars: Controlling Ring Dynamics in the Solid-State via a Reversible Phase Change. J. Am. Chem. Soc..

[47] Vukotic V.N., O’Keefe C.A., Zhu K., Harris K.J., To C., Schurko R.W., Loeb S.J. (2015). Mechanically Interlocked Linkers inside Metal–Organic Frameworks: Effect of Ring Size on Rotational Dynamics. J. Am. Chem. Soc..

[48] Farahani N., Zhu K., O’Keefe C.A., Schurko R.W., Loeb S.J. (2016). Thermally Driven Dynamics of a Rotaxane Wheel about an Imidazolium Axle inside a Metal-Organic Framework. Chempluschem.

[49] Karlen S.D., Garcia-Garibay M.A. (2005). yoAmphidynamic Crystals: Structural Blueprints for Molecular Machines. Mol. Mach..

[50] Wittebort R.J., Olejniczak E.T., Griffin R.G. (1987). Analysis of Deuterium Nuclear Magnetic Resonance Line Shapes in Anisotropic Media. J. Chem. Phys..

[51] Sutrisno A., Huang Y. (2013). Solid-State NMR: A Powerful Tool for Characterization of Metal-Organic Frameworks. Solid State Nucl. Magn. Reson..

[52] Henrichs P.M., Hewitt J.M., Linder M. (1984). Experimental Aspects of Deuterium NMR of Solids. J. Magn. Reson..

[53] Damron J.T., Ma J., Kurz R., Saalwächter K., Matzger A.J., Ramamoorthy A. (2018). The Influence of Chemical Modification on Linker Rotational Dynamics in Metal–Organic Frameworks. Angew. Chem. Int. Ed..

[54] Kremer F., Schönhals A. (2003). Broadband Dielectric Spectroscopy.

[55] Winston E.B., Lowell P.J., Vacek J., Chocholousová J., Michl J., Price J.C. (2008). Dipolar Molecular Rotors in the Metal-Organic Framework Crystal IRMOF-2. Phys. Chem. Chem. Phys..

[56] Devautour-Vinot S., Maurin G., Serre C., Horcajada P., Paula Da Cunha D., Guillerm V., De Souza Costa E., Taulelle F., Martineau C. (2012). Structure and Dynamics of the Functionalized MOF Type UiO-66(Zr): NMR and Dielectric Relaxation Spectroscopies Coupled with DFT Calculations. Chem. Mater..

[57] Frunza S., Schönhals A., Frunza L., Ganea P., Kosslick H., Harloff J., Schulz A. (2010). Molecular Relaxation Processes in a MOF-5 Structure Revealed by Broadband Dielectric Spectroscopy: Signature of Phenylene Ring Fluctuations. J. Phys. Chem. B.

[58] Balčiūnas S., Šimėnas M., Pavlovaitė D., Kinka M., Shieh F.-K., Wu K.C.-W., Banys J., Grigalaitis R. (2019). Low-Frequency Dipolar Dynamics and Atmospheric Effects in ZIF-90 Metal–Organic Framework. J. Phys. Chem. C.

[59] Greaves G.N., Meneau F., Majérus O., Jones D.G., Taylor J. (2005). Identifying Vibrations That Destabilize Crystals and Characterize the Glassy State. Science.

[60] Ryder M.R., Civalleri B., Bennett T.D., Henke S., Rudić S., Cinque G., Fernandez-Alonso F., Tan J.-C. (2014). Identifying the Role of Terahertz Vibrations in Metal-Organic Frameworks: From Gate-Opening Phenomenon to Shear-Driven Structural Destabilization. Phys. Rev. Lett..

[61] Tan N.Y., Ruggiero M.T., Orellana-Tavra C., Tian T., Bond A.D., Korter T.M., Fairen-Jimenez D., Axel Zeitler J. (2015). Investigation of the Terahertz Vibrational Modes of ZIF-8 and ZIF-90 with Terahertz Time-Domain Spectroscopy. Chem. Commun..

[62] Kuc A., Enyashin A., Seifert G. (2007). Metal−Organic Frameworks: Structural, Energetic, Electronic, and Mechanical Properties. J. Phys. Chem. B.

[63] Kolokolov D.I., Jobic H., Stepanov A.G., Plazanet M., Zbiri M., Ollivier J., Guillerm V., Devic T., Serre C., Férey G. (2010). Comparison of the Dynamics of MIL-53(Cr) and MIL-47(V) Frameworks Using Neutron Scattering and DFT Methods. Eur. Phys. J. Spec. Top..

[64] Burtch N.C., Torres-Knoop A., Foo G.S., Leisen J., Sievers C., Ensing B., Dubbeldam D., Walton K.S. (2015). Understanding DABCO Nanorotor Dynamics in Isostructural Metal–Organic Frameworks. J. Phys. Chem. Lett..

[65] Burtch N.C., Dubbeldam D., Walton K.S. (2015). Investigating Water and Framework Dynamics in Pillared MOFs. Mol. Simul..

[66] Namsani S., Yazaydin A.O. (2018). Electric Field Induced Rotation of Halogenated Organic Linkers in Isoreticular Metal–organic Frameworks for Nanofluidic Applications. Mol. Syst. Des. Eng..

[67] Haldoupis E., Watanabe T., Nair S., Sholl D.S. (2012). Quantifying Large Effects of Framework Flexibility on Diffusion in MOFs: CH_4_ and CO_2_ in ZIF-8. ChemPhysChem.

[68] Parkes M.V., Demir H., Teich-McGoldrick S.L., Sholl D.S., Greathouse J.A., Allendorf M.D. (2014). Molecular Dynamics Simulation of Framework Flexibility Effects on Noble Gas Diffusion in HKUST-1 and ZIF-8. Microporous Mesoporous Mater..

[69] Elsaidi S.K., Mohamed M.H., Simon C.M., Braun E., Pham T., Forrest K.A., Xu W., Banerjee D., Space B., Zaworotko M.J. (2017). Effect of Ring Rotation upon Gas Adsorption in SIFSIX-3-M (M = Fe, Ni) Pillared Square Grid Networks. Chem. Sci..

[70] Laio A., Gervasio F.L. (2008). Metadynamics: A Method to Simulate Rare Events and Reconstruct the Free Energy in Biophysics, Chemistry and Material Science. Rep. Prog. Phys..

[71] Vymětal J., Vondrášek J. (2010). Metadynamics As a Tool for Mapping the Conformational and Free-Energy Space of Peptides—The Alanine Dipeptide Case Study. J. Phys. Chem. B.

[72] Haigis V., Coudert F.-X., Vuilleumier R., Boutin A., Fuchs A.H. (2015). Hydrothermal Breakdown of Flexible Metal–Organic Frameworks: A Study by First-Principles Molecular Dynamics. J. Phys. Chem. Lett..

[73] Chen S., Lucier B.E.G., Boyle P.D., Huang Y. (2016). Understanding the Fascinating Origins of CO_2_ Adsorption and Dynamics in MOFs. Chem. Mater..

[74] Katsoulidis A.P., Antypov D., Whitehead G.F.S., Carrington E.J., Adams D.J., Berry N.G., Darling G.R., Dyer M.S., Rosseinsky M.J. (2019). Chemical Control of Structure and Guest Uptake by a Conformationally Mobile Porous Material. Nature.

[75] Yang W., Davies A.J., Lin X., Suyetin M., Matsuda R., Blake A.J., Wilson C., Lewis W., Parker J.E., Tang C.C. (2012). Selective CO_2_ Uptake and Inverse CO_2_/C_2_H_2_ Selectivity in a Dynamic Bifunctional Metal–organic Framework. Chem. Sci..

[76] Murdock C.R., McNutt N.W., Keffer D.J., Jenkins D.M. (2014). Rotating Phenyl Rings as a Guest-Dependent Switch in Two-Dimensional Metal–Organic Frameworks. J. Am. Chem. Soc..

[77] Hyun S., Lee J.H., Jung G.Y., Kim Y.K., Kim T.K., Jeoung S., Kwak S.K., Moon D., Moon H.R. (2016). Exploration of Gate-Opening and Breathing Phenomena in a Tailored Flexible Metal–Organic Framework. Inorg. Chem..

[78] Yue Y., Rabone J.A., Liu H., Mahurin S.M., Li M.-R., Wang H., Lu Z., Chen B., Wang J., Fang Y. (2015). A Flexible Metal–Organic Framework: Guest Molecules Controlled Dynamic Gas Adsorption. J. Phys. Chem. C.

[79] Sakaida S., Otsubo K., Sakata O., Song C., Fujiwara A., Takata M., Kitagawa H. (2016). Crystalline Coordination Framework Endowed with Dynamic Gate-Opening Behaviour by Being Downsized to a Thin Film. Nat. Chem..

[80] Serra-Crespo P., Gobechiya E., Ramos-Fernandez E.V., Juan-Alcañiz J., Martinez-Joaristi A., Stavitski E., Kirschhock C.E.A., Martens J.A., Kapteijn F., Gascon J. (2012). Interplay of Metal Node and Amine Functionality in NH_2_-MIL-53: Modulating Breathing Behavior through Intra-Framework Interactions. Langmuir.

[81] Banerjee D., Wang H., Plonka A.M., Emge T.J., Parise J.B., Li J. (2016). Direct Structural Identification of Gas Induced Gate-Opening Coupled with Commensurate Adsorption in a Microporous Metal-Organic Framework. Chem. A Eur. J..

[82] Bux H., Liang F., Li Y., Cravillon J., Wiebcke M., Caro J. (2009). Zeolitic Imidazolate Framework Membrane with Molecular Sieving Properties by Microwave-Assisted Solvothermal Synthesis. J. Am. Chem. Soc..

[83] Fairen-Jimenez D., Galvelis R., Torrisi A., Gellan A.D., Wharmby M.T., Wright P.A., Mellot-Draznieks C., Düren T. (2012). Flexibility and Swing Effect on the Adsorption of Energy-Related Gases on ZIF-8: Combined Experimental and Simulation Study. Dalt. Trans..

[84] Seo J., Matsuda R., Sakamoto H., Bonneau C., Kitagawa S. (2009). A Pillared-Layer Coordination Polymer with a Rotatable Pillar Acting as a Molecular Gate for Guest Molecules. J. Am. Chem. Soc..

[85] Zhang J.-P., Chen X.-M. (2009). Optimized Acetylene/Carbon Dioxide Sorption in a Dynamic Porous Crystal. J. Am. Chem. Soc..

[86] Zhang Z., Yang Q., Cui X., Yang L., Bao Z., Ren Q., Xing H. (2017). Sorting of C_4_ Olefins with Interpenetrated Hybrid Ultramicroporous Materials by Combining Molecular Recognition and Size-Sieving. Angew. Chemie Int. Ed..

[87] Andres-Garcia E., Oar-Arteta L., Gascon J., Kapteijn F. (2019). ZIF-67 as Silver-Bullet in Adsorptive Propane/propylene Separation. Chem. Eng. J..

[88] Hartmann M., Böhme U., Hovestadt M., Paula C. (2015). Adsorptive Separation of Olefin/Paraffin Mixtures with ZIF-4. Langmuir.

[89] Böhme U., Barth B., Paula C., Kuhnt A., Schwieger W., Mundstock A., Caro J., Hartmann M. (2013). Ethene/Ethane and Propene/Propane Separation via the Olefin and Paraffin Selective Metal–Organic Framework Adsorbents CPO-27 and ZIF-8. Langmuir.

[90] Luna-Triguero A., Vicent-Luna J.M., Calero S. (2018). Phase Transition Induced by Gas Adsorption in Metal-Organic Frameworks. Chem. A Eur. J..

[91] Zhang L., Jiang K., Jiang M., Yue D., Wan Y., Xing H., Yang Y., Cui Y., Chen B., Qian G. (2016). A Highly Stable Amino-Coordinated MOF for Unprecedented Block off N_2_ Adsorption and Extraordinary CO_2_/N_2_ Separation. Chem. Commun..

[92] Ania C.O., García-Pérez E., Haro M., Gutiérrez-Sevillano J.J., Valdés-Solís T., Parra J.B., Calero S. (2012). Understanding Gas-Induced Structural Deformation of ZIF-8. J. Phys. Chem. Lett..

[93] Kanoo P., Reddy S.K., Kumari G., Haldar R., Narayana C., Balasubramanian S., Maji T.K. (2012). Unusual Room Temperature CO_2_ Uptake in a Fluoro-Functionalized MOF: Insight from Raman Spectroscopy and Theoretical Studies. Chem. Commun..

[94] Gee J.A., Sholl D.S. (2016). Effect of Framework Flexibility on C_8_ Aromatic Adsorption at High Loadings in Metal–Organic Frameworks. J. Phys. Chem. C.

[95] Hong Y., Lam J.W.Y., Tang B.Z. (2011). Aggregation-Induced Emission. Chem. Soc. Rev..

[96] Qin A., Jim C.K.W., Tang Y., Lam J.W.Y., Liu J., Mahtab F., Gao P., Tang B.Z. (2008). Aggregation-Enhanced Emissions of Intramolecular Excimers in Disubstituted Polyacetylenes. J. Phys. Chem. B.

[97] Dong Y., Lam J.W.Y., Qin A., Liu J., Li Z., Tang B.Z., Sun J., Kwok H.S. (2007). Aggregation-Induced Emissions of Tetraphenylethene Derivatives and Their Utilities as Chemical Vapor Sensors and in Organic Light-Emitting Diodes. Appl. Phys. Lett..

[98] Shustova N.B., McCarthy B.D., Dincă M. (2011). Turn-On Fluorescence in Tetraphenylethylene-Based Metal–Organic Frameworks: An Alternative to Aggregation-Induced Emission. J. Am. Chem. Soc..

[99] Du T., Zhang H., Ruan J., Jiang H., Chen H.-Y., Wang X. (2018). Adjusting the Linear Range of Au-MOF Fluorescent Probes for Real-Time Analyzing Intracellular GSH in Living Cells. ACS Appl. Mater. Interfaces.

[100] Serra-Crespo P., Van Der Veen M.A., Gobechiya E., Houthoofd K., Filinchuk Y., Kirschhock C.E.A., Martens J.A., Sels B.F., De Vos D.E., Kapteijn F. (2012). NH2-MIL-53(Al): A High-Contrast Reversible Solid-State Nonlinear Optical Switch. J. Am. Chem. Soc..

[101] Balestra S.R.G., Bueno-Perez R., Hamad S., Dubbeldam D., Ruiz-Salvador A.R., Calero S. (2016). Controlling Thermal Expansion: A Metal–Organic Frameworks Route. Chem. Mater..

[102] Collings I.E., Tucker M.G., Keen D.A., Goodwin A.L. (2014). Geometric Switching of Linear to Area Negative Thermal Expansion in Uniaxial Metal–organic Frameworks. CrystEngComm.

[103] Lock N., Wu Y., Christensen M., Cameron L.J., Peterson V.K., Bridgeman A.J., Kepert C.J., Iversen B.B. (2010). Elucidating Negative Thermal Expansion in MOF-5. J. Phys. Chem. C.

[104] Lock N., Christensen M., Kepert C.J., Iversen B.B. (2013). Effect of Gas Pressure on Negative Thermal Expansion in MOF-5. Chem. Commun..

[105] Peterson V.K., Kearley G.J., Wu Y., Ramirez-Cuesta A.J., Kemner E., Kepert C.J. (2010). Local Vibrational Mechanism for Negative Thermal Expansion: A Combined Neutron Scattering and First-Principles Study. Angew. Chem. Int. Ed..

[106] Wang L., Wang C., Sun Y., Shi K., Deng S., Lu H. (2016). Large Negative Thermal Expansion Provided by Metal-Organic Framework MOF-5: A First-Principles Study. Mater. Chem. Phys..

[107] Pang J., Liu C., Huang Y., Wu M., Jiang F., Yuan D., Hu F., Su K., Liu G., Hong M. (2016). Visualizing the Dynamics of Temperature- and Solvent-Responsive Soft Crystals. Angew. Chem. Int. Ed..

